# Using Clustering, Statistical Modeling, and Climate Change Projections to Analyze Recent and Future Region‐Specific Compound Ozone and Temperature Burden Over Europe

**DOI:** 10.1029/2021GH000561

**Published:** 2022-04-16

**Authors:** Sally Jahn, Elke Hertig

**Affiliations:** ^1^ Regional Climate Change and Health Institute of Geography and Faculty of Medicine University of Augsburg Augsburg Germany; ^2^ Regional Climate Change and Health Faculty of Medicine University of Augsburg Augsburg Germany

**Keywords:** air pollution, air temperature, climate change, compound events, spatial clustering, statistical downscaling

## Abstract

High ground‐level ozone concentrations and high air temperatures present two health‐relevant natural hazards. The most severe health outcomes are generally associated with concurrent elevated levels of both variables, representing so‐called compound ozone and temperature (o‐t‐) events. These o‐t‐events, their relationship with identified main meteorological and synoptic drivers, as well as ozone and temperature levels themselves and the linkage between both variables, vary temporally and with the location of sites. Due to the serious health burden and its spatiotemporal variations, the analysis of o‐t‐events across the European domain represents the focus of the current work. The main objective is to model and project present and future o‐t‐events, taking region‐specific differences into account. Thus, a division of the European domain into six o‐t‐regions with homogeneous, similar ground‐level ozone and temperature characteristics and patterns built the basis of the study. In order to assess region‐specific main meteorological and synoptic drivers of o‐t‐events, statistical downscaling models were developed for selected representative stations per o‐t‐region. Statistical climate change projections for all central European o‐t‐regions were generated to assess potential frequency shifts of o‐t‐events until the end of the 21st century. The output of eight Earth System Models from the sixth phase of the Coupled Model Intercomparison Project considering SSP245 and SSP370 scenario assumptions was applied. By comparing midcentury (2041–2060) and late century (2081–2100) time slice differences with respect to a historical base period (1995–2014), substantial increases of the health‐relevant compound o‐t‐events were projected across all central European regions.

## Key Points

- A clustering approach based on ground‐level ozone and air temperature leads to a division of Europe into six ozone and temperature regions
- Statistical downscaling models identify region‐specific main meteorological and synoptic drivers of compound ozone and temperature events
- Climate change projections point to an increase in these compound events until the end of the 21st century in central Europe

## 1 Introduction

Simultaneous occurrences of elevated levels of tropospheric, ground‐level ozone (O_3_) concentrations, and surface air temperatures can be defined as so‐called compound events (Zscheischler et al., 2020). O_3_ and air temperatures are often strongly correlated and elevated levels repeatedly coincide. Their relationship and joint occurrences have already presented the focus of a variety of recent health‐related environmental studies (e.g., Abdullah et al., 2017; Hertig, 2020; Schnell & Prather, 2017). There is evidence that the co‐occurrence of both variables results in a human health risk beyond the sum of their individual effects (Hertig et al., 2020; Katsouyanni & Analitis, 2009; Pattenden et al., 2010).

The variations, linkage, and co‐occurrences of O_3_ and air temperatures are based on the specific nonlinear, temperature‐dependent and sunlight‐driven chemistry of ozone formation determined by the overall ambient, photochemical conditions and precursor emissions (Pusede et al., 2015). O_3_ is influenced by a large variety of, often also temperature‐dependent and partly interacting, chemical‐based factors (e.g., composition of VOCs or the cycle and supply of odd hydrogen radicals). O_3_ is also strongly dependent on meteorological variables and synoptic conditions (please refer to Sillman, 1999; Sillman, 2003 for more background information on ozone formation). Porter and Heald (2019) analyzed the summertime ozone‐temperature (o‐t‐) relationship in the United States and Europe. They found that a high portion of the o‐t‐correlation can be attributed to four temperature‐dependent, chemistry‐based mechanisms (i.e., soil NO_x_ and biogenic VOC emissions, PAN dissociation and O_3_ dry decomposition). But, especially in Europe, the correlation is linked to meteorological phenomena. Consequently, ozone and air temperature levels, their linkage, and respective compound events can be expected to differ regionally across Europe.

Recent work confirms that the linkage between ozone and temperature must be evaluated over a broad range of temperatures. Especially, ozone levels in polluted regions show strong dependences on temperature. Elevated levels are generally associated with air temperatures surpassing 20°C (Sillman, 2003). Steiner et al. (2010) analyzed the o‐t‐relationship in four air basins in California. They found an approximately linear increase in maximum ground‐level ozone concentrations between 295 and 312 K (about 22–37°C), but they also provided evidence that ozone may plateau or decrease under extremely high temperatures (>37°C). This is due to a decrease in PAN and a reduction of isoprene emissions at these high‐temperature levels. Varotsos et al. (2019) confirmed by their analysis of two major European heatwave episodes (2003 and 2014) that higher O_3_ concentrations are in general accompanied by higher air temperatures. However, a break of the o‐t‐relationship in the higher percentiles of both variables was present during the 2003 heatwave. This was mainly due to reduced biogenic emissions under very high temperatures in southern parts of their European study domain. The linkage between both variables under current and future emission and climatic conditions has been the focus of further studies (e.g., Bloomer et al., 2009; Coates et al., 2016; Colette et al., 2015; Rasmussen et al., 2013). In previous work from Jahn and Hertig (2021), co‐occurring elevated O_3_ and air temperature levels in Europe were considered. A strong o‐t‐correlation accompanied by a high frequency of concurrent high levels of both variables becomes evident in central Europe (i.e., Austria, Belgium, Germany, The Netherlands, and Switzerland). However, a strong o‐t‐relationship is not observable in more northern‐ and southern‐located stations (i.e., in Great Britain, Greece, Finland, and Sweden). These findings also suggest that the o‐t‐linkage regionally varies across Europe.

Considering the meteorological and synoptic conditions influencing compound o‐t‐events, various previous studies exist focusing on the identification of respective mechanisms affecting ozone alone or in combination with air temperature levels (e.g., Hertig, 2020; Jahn & Hertig, 2021; Kerr et al., 2019). Regional variations have already become evident. For example, Otero et al. (2016) found that over central Europe meteorological variables play an important role as drivers of maximum daily ozone and its extreme values. However, in northern and southern parts of the study domain, the influence of ozone persistence and hence precursor emissions is comparably strong on ozone exceedances. These results suggest a stronger role of preceding ozone conditions compared to meteorological variables in northern and southern Europe. Hertig et al. (2020) analyzed single and concurrent heat and ozone waves in two climatically different regions, Portugal and Bavaria. They found that for Portugal, representative for southern Europe, synoptic circulation weather types (WT), promoting the inflow of hot and dry air masses, and the advection of ozone and its precursors from remote areas highly influence observed O_3_ levels.

One way of addressing regional differences consists of dividing the European domain into regions of coherent spatiotemporal o‐t‐characteristics and patterns, which may be affected by different main drivers under climatic and environmental changes. Regionalizations, comprising the classification of sites and resulting in the spatial division into zones or regions, have been generated in previous studies based on ozone (Boleti et al., 2020; Carro‐Calvo et al., 2017; Lehman et al., 2004; Lyapina et al., 2016; Varotsos et al., 2013) or temperature (Bador et al., 2015; Chidean et al., 2015; Scotto et al., 2011). However, to the best of our knowledge, a spatial clustering based on both target variables over Europe in order to account for recent and future spatiotemporal variations has not been conducted so far.

The present study fills this gap by presenting a regionalization approach based on O_3_ and air temperatures to account for spatiotemporally varying environmental and climatic conditions across Europe. Additionally, regionally different recent and future health burden over Europe is highlighted. The definition of robust, geographically, and environmentally meaningful European o‐t‐regions using a clustering approach constitutes a novelty and builds the basis of the presented work. Based on the identified o‐t‐regions, statistical downscaling models of health‐relevant compound o‐t‐events were generated. Only distinct meteorological variables and synoptic conditions that carry physically meaningful and relevant information were considered. WT to analyze synoptic conditions has been applied in various recent air quality studies already (e.g., Otero et al., 2016; Russo et al., 2015). In this study, Self‐Organizing Maps (SOM), an artificial neural network algorithm based on unsupervised learning, was used. SOM has been applied within a climatic or synoptic context in several existing studies (e.g., Gibson et al., 2017; Odoulami et al., 2020; Sheridan & Lee, 2011; Tian et al., 2014; Vesanto & Alhoniemi, 2000). Region‐specific projections based on statistical downscaling models using the climate change scenario assumptions SSP245 and SSP370 until the end of the 21st century based on the updated Coupled Model Intercomparison Project Phase 6 (CMIP6) were generated.

The main contributions of the presented work are hence the following: first, the identification of six European o‐t‐regions highlighting the regional phenomena of o‐t‐characteristics, patterns, and variabilities. Second, the specification and comparison of region‐specific variations of o‐t‐relationships. Third, statistical models and downscaling climate change projections of compound o‐t‐events, including the evaluation of the suitability of the applied study framework for all central European o‐t‐regions.

The remainder of this paper is structured as follows. Section 2 presents the initial selection and preprocessing of station‐based ozone and temperature data and the application of the regionalization process. Section 3 introduces the preparation of predictands as well as the selection and prepossessing of predictor data. All applied statistical models and projection approaches are presented in Section 4. Section 5 summarizes the main contributions with discussion and limitations presented in Sections 6 and 7. Section 8 contains concluding remarks. In the Table S1 in Supporting Information S1, an overview of all abbreviations and acronyms used in this paper is provided.

## 2 Station‐Based Data and Regionalization

### 2.1 Data and Station Selection

This section describes the collection, preprocessing, and selection of European stations, providing measurement data on surface maximum daily ozone and air temperature. Ozone and temperature stations were paired based on the location of sites and on the extracted and processed observation data. Station pairs represented the final locations of combined ozone and temperature observations.

#### 2.1.1 Data Sources

The processing and preselection of daily maximum surface air temperature (TX) and O_3_ data were conducted to generate a homogenized database with consistently submitted and prepared air quality and temperature monitoring data. Station‐based, valid TX data based on the available adjusted, homogenized blended temperature series of the European Climate Assessment and Data set project (ECA&D) (Klein Tank et al., 2002) were obtained. Air Quality eReporting ozone pollution data from the European Environment Agency with an originally reported hourly time resolution were extracted (EEA, 2017). Daily 8‐hr running means and subsequently daily maximum 8‐hr running means (MDA8O3) were calculated by only considering valid labeled hourly measurements. Valid MDA8O3 data finally used for further analysis were only calculated if at least 18 valid 8‐hr running means were available for a particular day. In order to achieve a suitable and sufficient coverage with ozone stations across the whole European domain, the years from 2004 to 2018 built the temporal focus. This specific period of time is hereinafter referred to as base period. To reduce the direct influence of emissions of precursors and hence reactive pollutants affecting ozone formation and depletion processes, only urban, suburban, and rural background stations (hereinafter referred to as station types) were incorporated in the analysis. In general, only ozone and temperature stations providing data for each year in the base period were selected. If not otherwise specified, temperature or TX as well as ozone or MDA8O3 refer to the finally approved station‐based daily maximum air temperature and ozone values. All further analyses were based on the o‐t‐season from April to September.

#### 2.1.2 Station Pairs

Station pairs, combining temperature and ozone measurements, were built assigning a temperature station with a maximum distance of 15 km and a maximum altitude difference of 200 m to each ozone station. In order to minimize the influence of missing values, station pairs with daily data for both ozone and temperature with a data coverage of more than 75% in the o‐t‐season from 2004 to 2018 were selected. If another ozone station matched with the same temperature station, only the station pair with an overall higher data coverage was kept for further analysis to avoid the occurrence of the same temperature data series for different ozone locations. As a result, 161 station pairs represent the final database. The mean distance between all paired stations is 5.39 km (min 0.09 km, max 14.99 km, and median 4.45 km) and the mean altitude difference is 28.47 m (min 0 m, max 189 m, and median 13 m). An overview of all chosen station pairs with more detailed station metadata and characteristics is available in the Table S2 in Supporting Information S1. Hereinafter, the linked station pairs are simplified referred to as station. All spatial station information is represented by the respective station pair's ozone site. Station altitudes in the final database vary between min −1.0 m and max 3,106.0 m (mean 219.93 m and median 111.0 m).

### 2.2 Regionalization

The overall goal of the regionalization is a spatial division of the study domain into distinct o‐t‐regions. A common hierarchical clustering technique based on MDA8O3 and TX conditions in Europe is applied to produce stable clusters, that is, the final o‐t‐regions.

#### 2.2.1 Ozone and Temperature Regions

Regionalization aims to generate homogenous regions, showing the highest dissimilarities between each other. An agglomerative hierarchical clustering approach, which is an unsupervised learning algorithm, was applied to primarily account for region‐specific temporal variabilities like day‐to‐day variations in ozone and air temperatures. The algorithm builds nested clusters by merging them successively based on the computation of distances. The Ward variance minimization algorithm (hereinafter referred to as Ward's method) was selected out of a large variety of possible linkage methods. Ward's method not only relies on a simple (Euclidean) distance calculation, but also accounts for cluster variances by minimizing the total within‐cluster and maximizing the between‐cluster variance. The separability of clusters can finally be clearly measured by Ward's distance (e.g., Backhaus et al., 2016; Wilks, 2006). In future work, new instances can be easily assigned to a generated cluster by simply calculating and minimizing Ward's distance. This represents a significant advantage if, for example, the database is expanded by additional stations within the given European domain. Ward's method also shows the most promising results regarding the generation of geographically and environmentally meaningful regions with distinct o‐t‐characteristics and patterns (please refer to Section 5 for details).

The selection of the optimum number of clusters is generally challenging and several metrics can be applied. In this study, the overall goal is to find distinct o‐t‐regions, which yield a balance between a sufficient number of regions to explain spatiotemporal variability while still providing a reasonable degree of generalization. A minimum of at least four distinct o‐t‐regions to account for general geographical differences was thus chosen. In this context, besides a manual evaluation, the within‐cluster similarity and thus distances and height calculations based on Ward's method were considered. Primarily, the dendrogram as well as the elbow method were used to define the optimum number of clusters.

Station‐based MDA8O3 and TX data were separately preprocessed by filling missing values with daily means in order to perform the cluster procedure. Considering joint TX and MDA8O3 time series data for clustering, the station‐based data were at first standardized by removing the mean to center the data and by scaling it to unit variance. Standardization in this study was always conducted following this procedure.

#### 2.2.2 Representative Stations

Representative stations were defined for each region to build statistical downscaling models valid for the whole o‐t‐region. The stations were selected based on the Euclidean distance of a station to its respective cluster centroid. While the regionalization mainly aims to group similar daily variability, absolute station‐based values may highly differ within an o‐t‐region. This may be particularly true between different site‐specific station characteristics. If available, one urban, suburban, and rural representative stations per region were hence selected. Thus, MDA8O3‐ and TX‐level differences per station type in an o‐t‐region and so intraregional differences were accounted for by selecting more than one representative station per region. Even if all stations of a region vary similarly and variances of each cluster were minimized by the chosen clustering procedure, type‐specific ozone, and temperature‐level variations were hence be represented in subsequent modeling and projection processes. The hypothesis that compound o‐t‐events in each region depend on the same main drivers could thus be tested and possible different future sensitivities under future climate change were accounted for.

## 3 Predictor and Predictand Data

### 3.1 Compound Events

Health‐relevant, concurrent high levels of MDA8O3 and TX were used to define o‐t‐events regarding each specific representative station. For MDA8O3, pollution levels surpassing a threshold of 100 μg/m³ determined an ozone event. The threshold is in accordance with recommended standards of the World Health Organization (WHO, 2006, 2021). A local and station‐specific percentile‐based approach was chosen for TX to define non‐optimum ambient temperatures per site. Based on a 31‐day window, the 80^th^ percentiles (similar to e.g., Della‐Marta et al., 2007) were calculated for each individual day based on the respective daily TX values across all years in the base period (hereinafter referred to as ^80^TX). This uniform 80^th^ percentile value for all regions and stations eases the comparability and interpretation of o‐t‐events across the whole study domain. Additionally, the station‐based approach regards the fact that individuals generally adapt to their local weather conditions. So, spatial but also seasonal acclimatization processes as well as, to some extent, location‐based (population) characteristics are taken into account. If an o‐t‐event was observed, temperature levels as well as ozone concentrations are needed to exceed these predefined thresholds on the same day. Compound events were in general coded as 1 (event) and 0 (nonevent) in statistical models and climate change projections.

### 3.2 ERA5 Reanalysis Data

The selection of suitable variables to predict compound o‐t‐events was primarily determined by an analysis and a literature review (e.g., Hertig et al., 2019; Jahn & Hertig, 2021; Otero et al., 2016), data availability in the chosen ERA5 reanalysis, as well as Earth System Models (ESM). Predictor metrics used in this study are hence split into two types, meteorological predictors and synoptic WT. Even if nonphysical predictors may provide an additive value for predictions, further metrics like persistence of ozone or the inclusion of harmonic functions to assess regional variations of the seasonal cycle were not included in the statistical models as they do not represent direct physical drivers of ozone and temperature.

#### 3.2.1 Meteorological Predictors

Meteorological predictors were selected to include one circulation dynamic, humidity, thermal, and radiation‐based predictor to cover each climate change‐relevant information. Simultaneously, overfitting in the modeling process by not including several predictors of almost the same information was avoided. The following meteorological variables provided by the ERA5 reanalysis data set from the European Centre for Medium‐Range Weather Forecasts (Hersbach & Dee, 2016) were retrieved (units in square brackets): geopotential heights (GH) (m), specific humidity (SH) (kg/kg), and mean air temperatures (MT) (°C). The predictors were all downloaded at the 850 hPa level for the whole European domain (25°N–70°N, 40°E−25°W) with a 1 × 1° resolution. Hence, predictor data approximately directly above the boundary layer not being strongly influenced by varying orographic and topographic conditions were selected. Furthermore, surface solar radiation downward (SSRD) was extracted (W/m^2^). For the purpose of including for each specific location the best‐fitting predictor data, the mean of the nine grid boxes covering the area over and around each representative site was used to define station‐based daily predictor time series. The daily meteorological predictor time series were all standardized before entering the statistical modeling process.

The agreement of predictors across all stations of one o‐t‐region was evaluated by manual inspection and correlation analysis. Similar monthly meteorological conditions with comparable variations were observable within a region in the o‐t‐seasons from 2004 to 2018. Spearman rank correlation coefficients of at least 0.5, by considering daily pairwise station‐based predictor time series within a region, were computed. In summary, the presented meteorological information of each representative station fits quite well its overall region conditions. The same predictors show similar characteristics and variations and are highly correlated across all stations of an o‐t‐region.

#### 3.2.2 Synoptic Weather Types

Daily ERA5 mean sea level pressure (MSLP) was used to classify synoptic WT, representing the second group of predictors. The data were extracted with the same spatial resolution and coverage as the meteorological predictors. SOM was applied on the preprocessed European MSLP data. WT patterns commonly show the MSLP—“weight” of each node of the generated SOM grid.

In general, a SOM grid needs to be defined by size and hence number of nodes. A balance must be found between a too high complexity of the model and choosing enough nodes to represent all needed information. With a too small grid size, the output patterns are too general and important differences are missed. If the grid size is too large, the differences between neurons are too detailed. In projection studies, a not too high number of nodes with a more even distribution across all WT are favorable to avoid a significant number of “empty” nodes when applying ESM data.

In this study, the classification comprises all months in the o‐t‐season from 2004 to 2018. The WT are based on a rectangular SOM grid. Several grid sizes were tested within the classification from a minimum of four to a maximum of 20 WT. The topographic product as a measure for the degree of topology preservation was used to define an appropriate grid size. This metric can be combined with a detailed, far‐reaching hyperparameter tuning. Hyperparameter tuning was conducted by adjusting the parameters sigma, learning rate, and iterations of the SOM algorithm.

New samples are in general classified by evaluating the Euclidean distance between the sample and the weights of all nodes. Accordingly, WT were assigned to each day in the o‐t‐season from 2004 to 2018 by evaluating the Euclidean distance between each daily MSLP field and the weights of all nodes. A respective synoptic WT predictor time series based on numerical codes was hence created, later used for model building.

### 3.3 Earth System Model Data

For climate change projections, predictor data were extracted for the European study domain using output from eight CMIP6‐ESM (Eyring et al., 2016): BCC‐CSM2‐MR, CanESM5, FGOALS‐g3, INM‐CM5‐0, IPSL‐CM6A‐LR, MIROC6, MPI‐ESM1‐2‐HR, and MRI‐ESM2‐0. Historical ESM runs from 1995 to 2014 as well as SSP245 and SSP370 scenario runs from 2015 to 2100 (e.g., Gidden et al., 2019; O'Neill et al., 2016; Turnock et al., 2020) were downloaded under r1i1p1f1 initial conditions in a daily temporal resolution. The spatial resolution of the ESM data varies from 0.9° (MPI‐ESM1‐2‐HR) to more than 2.5° (CanESM5). Consequently, model data were regridded by bilinear interpolation to match the ERA5 resolution. Station‐specific meteorological time series were generated in accordance with the reanalysis predictor data by considering nine grid‐cell means. A specific WT was assigned to each day in the historical ESM as well as for both scenarios in accordance with the approach used in reanalysis. Thus, for each chosen ESM, daily WT time series consisting of numerical codes were generated by the evaluation of Euclidean distances.

A simple linear scaling bias correction technique, also used by for example, Gohar et al. (2017) and Teutschbein and Seibert (2012), was chosen and adapted to remove the monthly mean bias between climate model data and reanalysis. The monthly mean difference between station‐based meteorological reanalysis and historical ESM data was used to bias‐correct the respective ESM data. Based on the two‐sample Kolmogorov‐Smirnov test, the distributional similarity between meteorological ERA5 and monthly bias‐corrected ESM data was considered. The evaluation was conducted for each predictor variable across all representative stations and ESM. ESM and ERA5 predictor data show in general a high consistency across all ESM and variables with significant distributional differences only present for 12 ERA5‐ESM pairs (please refer to Table S3 in Supporting Information S1 for details). Thus, the ESM meteorological predictors are suitable to exchange the ERA5 predictor data to generate projections. For MSLP data, the bias correction was first conducted based on every grid cell in the European domain before the respective WT time series was generated. An overview and comparison of the occurrences of WT considering all eight ESM as well as ERA5 reanalysis data is given in Table S4 in Supporting Information S1.

ESM data were standardized before entering projections. Hereby, historical and one scenario, SSP245 or SSP370, time series were first merged and subsequently standardized with respect to their means and standard deviations. Consequently, variability and mean changes per scenario assumption could be evaluated.

## 4 Statistical Models and Climate Change Projections

### 4.1 Statistical Downscaling Models

Statistical models to establish the relationships between meteorological and synoptic conditions and o‐t‐events were generated. All representative stations of each individual o‐t‐region were considered. A station‐based approach with an individualized predictor screening process was chosen to assess suitable station‐specific predictors. Thus, the identification of specifics and differences within as well as between o‐t‐regions was simplified. Logistic regression (LR) was used to assess the likelihood of o‐t‐events. Site‐specific main drivers were identified. Statistical downscaling models were evaluated by considering model fit and performance.

#### 4.1.1 Logistic Regression

A specific type of generalized linear models, LR, was used to assess the probability of threshold exceedances, hence o‐t‐events. By applying LR, a sufficient number of events with respect to the number of selected predictors is generally needed. Even if, as rule of thumb, a common ratio of 10–20 events per variable is often used, existing studies suggest and apply a smaller ratio of 5–10 (Otero et al., 2016; Peduzzi et al., 1996). For modeling, strong imbalanced data sets with an unequal distribution of majority and minority classes (here o‐t‐events) are unfavorable. In this study, the minority class represents under 20% of all data points across all representative stations. Thus, data were resampled using the SMOTE (Synthetic Minority Oversampling Technique) algorithm. SMOTE creates synthetic samples from the minority class to get equally distributed data sets. At first, a station model was built on all available predictor data. The significance of a predictor was tested on the 95% significance level by applying Wald's Test. If the p‐value of a predictor exceeded 0.05, the predictor was excluded, and the station model was generated again based on the new subset of predictors. The process was repeated until only significant predictors entered the final station model.

A special focus in the presented work is set on the identification of the most important (MID), second (SMID) and third most important drivers (TMID) of o‐t‐events. In the statistical models, a predictor variable's effect on prediction can be expressed by its regression coefficient in terms of the log‐odds. If a predictor variable's effect on prediction is significantly different from zero, it contributes to the prediction of o‐t‐events. Leading drivers of o‐t‐events are identified by the strength of the relationship between each predictor and the target variable, given by the absolute value and the sign of its standardized regression coefficient. Predictor variables can hence be ranked by importance to identify station‐based main drivers.

#### 4.1.2 Model Fit and Performance

McFadden's Pseudo‐R^2^ (MF‐R^2^) based on the maximum likelihood concept is a common metric applied to assess model fit considering LR. By comparing two models, the one with higher MF‐R^2^ values represents generally a better‐fitted model. Common performance metrics with respect to imbalanced data sets in machine learning are Precision (P), Recall (R), and the F1‐Score (He & Ma, 2013), all applied for model evaluation. All metrics indicate performance by values between zero and one with higher values showing a better model performance. The performance evaluation of the final station‐based LR models was embedded in a tenfold cross‐validation procedure. In general, a modeled probability value of 0.5 is often used to differ between event and nonevent days. To ensure a more balanced relationship between the fraction of actual observed events that were correctly predicted (P) and the fraction of the predicted events that did actually occur (R), a suitable probability threshold value was evaluated within an own, upstream tenfold cross‐validation procedure. The threshold for which P and R were closest to each other was selected. This model‐specific threshold was used in performance evaluation as well as for projections.

### 4.2 Climate Change Projections

Statistical climate change projections of compound o‐t‐events under future scenario assumptions were generated by replacing reanalysis with corresponding, preprocessed ESM predictor data in the final station‐specific statistical downscaling models. Only representative stations in regions having shown their suitability to project o‐t‐events under future scenario assumptions were incorporated. Thus, all regions with stations not showing a substantial, positive linkage between MDA8O3 and TX levels as well as a strong relationship of o‐t‐events and selected predictors were excluded from further analysis. Projected changes (%) and thus potential frequency shifts of o‐t‐event occurrences until the end of the 21st century were assessed by comparing time slice differences with respect to the number of o‐t‐event days. Potential frequency shifts were evaluated by comparing midcentury (2041–2060) and late century (2081–2100) time windows to a historical ESM period (1995–2014). Multi‐model (ensemble) means based on the climate change signal from eight CMIP6‐ESM were considered.

## 5 Results

### 5.1 Present‐Day Ozone and Temperature

The regionalization led to a division of the study domain in six o‐t‐regions. Representative stations were selected for each region. Region‐ and site‐specific characteristics as well as relationships between MDA8O3 and TX were analyzed. An in‐depth evaluation and interpretation of respective results are presented subsequently in Section 6.

#### 5.1.1 Ozone and Temperature Regions

**Figure 1:**
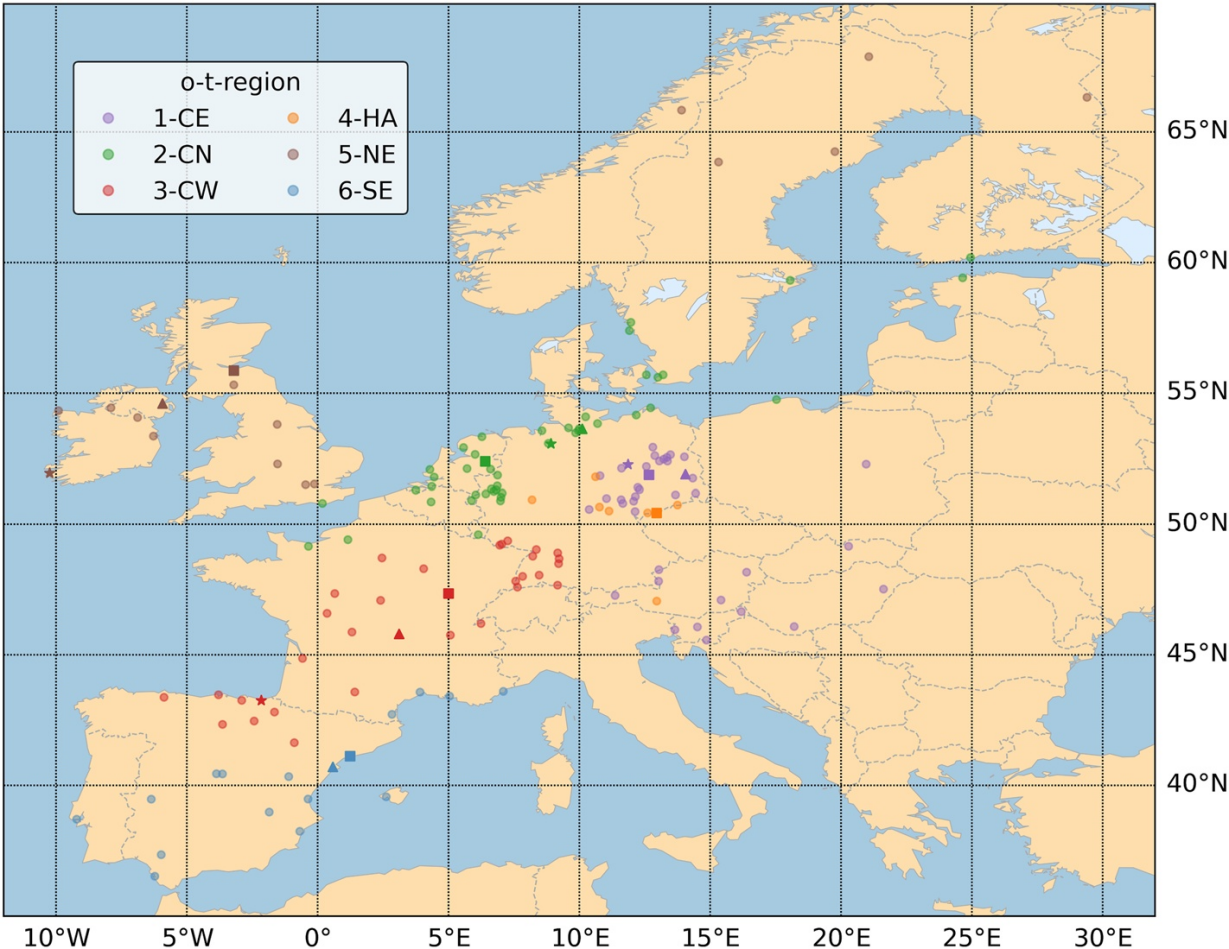
Location of all 161 stations in Europe. Color of points indicate to which of the following o‐t‐region each station is assigned: 1‐CE (**C**entral **E**astern), 2‐CN (**C**entral **N**orthern), 3‐CW (**C**entral **W**estern), 4‐HA (Central **H**igh **A**ltitude), 5‐NE (**N**orthern **E**uropean), and 6‐SE (**S**outhern **E**uropean o‐t‐region). Representative stations are highlighted by shape (squares = first, triangles = second, stars = third representative station) and color saturation.

The regionalization resulted in the following northern, southern, and four central European o‐t‐regions: 1‐CE (**C**entral **E**astern), 2‐CN (**C**entral **N**orthern), 3‐CW (**C**entral **W**estern), 4‐HA (Central **H**igh **A**ltitude), 5‐NE (**N**orthern **E**uropean), and 6‐SE (**S**outhern **E**uropean o‐t‐region). The location of all stations used in the cluster analysis and their assignments to each o‐t‐region are highlighted in Figure 1. In addition, Table 1 gives an overview of region‐specific ozone and temperature characteristics. Not taking 4‐HA into account, Table 1 reveals that the highest and lowest mean MDA803 and TX values are observable in 5‐NE and 6‐SE, located at the northern and southern rims of the study domain. The lowest, medium, and highest daily TX levels are generally accompanied by the lowest, medium, and highest daily MDA8O3 concentrations, not considering 4‐HA.

**Table 1:** Region‐Specific Ozone and Temperature Characteristics

O‐t‐region	Altitude	MDA8O3	MDA8O3	MDA8O3	TX	TX	TX
	mean/median	mean	median	25^th^/75^th^	mean	median	25^th^/75^th^
1‐CE	205.67/140.00	97.58	95.44	79.37/114.00	21.75	21.90	17.80/25.80
2‐CN	37.86/19.00	85.94	82.55	69.00/98.24	19.78	19.80	16.50/23.10
3‐CW	273.94/275.00	96.24	93.20	78.20/111.00	22.68	22.60	18.80/26.60
4‐HA	1205.13/916.5	105.86	104.00	87.75/122.40	13.41	14.10	9.00/18.60
5‐NE	170.59/130.00	71.02	69.40	57.00/84.16	16.28	16.70	13.40/19.50
6‐SE	236.88/81.00	105.85	104.00	91.00/119.00	27.17	27.50	23.40/31.00

*Note*. MDA8O3 (in μg/m^3^) and TX (in °C) characteristics are based on the o‐t‐season from 2004 to 2018. Mean, median, as well as 25th and 75th percentile values for each region are shown. Additionally, the mean and median altitude levels (in m) are depicted. Numbers are calculated on the basis of all available stations of an o‐t‐region.

Central European o‐t‐region 4‐HA is geographically scattered. The region contains mainly higher altitude stations with mean and median levels of 1205.13 m and 916.5 m (min 641 m and max 3,106 m), with two stations being located above 1000 m (representative station DESN053 and DEST039) and only one above 2,000 m (AT0SON1). Stations of 4‐HA show comparably low TX next to high MDA8O3 values due to their outstanding location characteristics, that is, high‐altitude levels determine relatively cold air temperatures, and stratospheric influences often affect daily ozone concentration levels. The separate clustering of these stations due to their deviant environmental and air pollution conditions is hence reasonable, outlined more in detail in the subsequent section and in Section 6.

**Figure 2:**
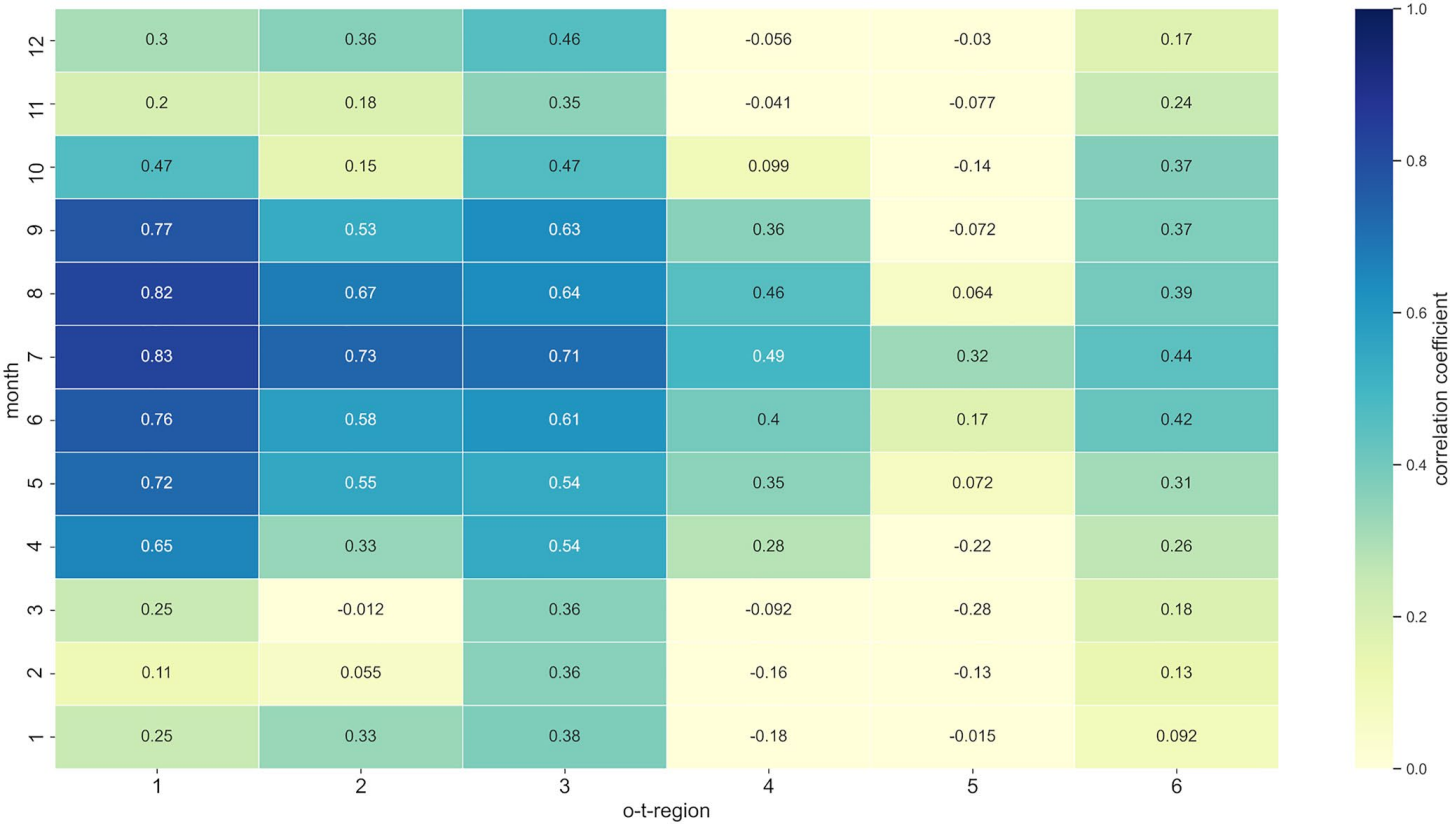
Spearman rank correlation coefficients between daily MDA8O3 concentrations and TX values for each o‐t‐region in Europe from 2004 to 2018. O‐t‐regions are specified by their respective numbers and depicted on the *x* axis. Months are shown as numbers (1 = January to 12 = December) on the *y* axis.

Figure 2 provides the main information that a strong linkage of ozone and temperature is primarily present in central Europe. Very strong relationships between MDA8O3 concentrations and TX values in the o‐t‐season become mainly apparent for 1‐CE, 2‐CN, and 3‐CW. Regions 5‐NE and 6‐SE, representing comparably low‐ and high‐temperature areas, show in general lower correlation coefficients. This is also evident for the o‐t‐season from April to September.

If site‐specific ozone chemistry characteristics highly determine ozone levels and related o‐t‐relationships, the observed deviant correlation levels could be associated with the varying number of stations per station type in each o‐t‐region. For example, 2‐CN consists of 61% urban stations, while only 35% of all stations in 5‐NE are urban (please refer to Table S5 in Supporting Information S1 for details). The different number of urban stations hence might affect the presented region‐specific o‐t‐correlations of 2‐CN and 5‐NE shown in Figure 2. Respective possible relationships were tested and could be ruled out by the analysis. Hence, station‐type influences do not generally determine region‐specific o‐t‐correlations.

The same evaluation as shown in Figure 2 was conducted on a station level. Monthly spearman rank correlation coefficients were calculated for each individual station of an o‐t‐region. The station‐specific monthly o‐t‐linkage from January to December based on all years from 2004 to 2018 was evaluated. The results were in accordance with the patterns shown in Figure 2. For the vast majority of stations, similar o‐t‐correlations as for the respective overall o‐t‐region could be observed. Considering 4‐HA, a high correlation of at least 0.6 for each month in the o‐t‐season becomes apparent for all stations but one (AT0SON1). AT0SON1 shows deviant o‐t‐correlations with coefficients of 0.4 or higher only computed for the months from June to August. AT0SON1 is located above 3,000 m, so it is primary installed to monitor background free‐tropospheric ozone concentrations. But as in three summer months, the o‐t‐correlation is still substantially observable, and to maintain consistency in the approach, AT0SON1 is kept in the database. Nevertheless, all subsequent results for 4‐HA can only partly be transferred to station AT0SON1.

#### 5.1.2 Representative Stations

Fifteen representative stations were extracted. The analysis revealed that the proximity of a station to its centroid, based on Euclidean distances, is in general independent of station types, including all representative stations. All but one representative station (ES1599 A representing the only rural station in 3‐CW) are nearby their respective cluster centroids. Representative stations are also highlighted and further described in Table S2 and S5 in Supporting Information S1. Not taking 4‐HA into account, the highest numbers of o‐event days are visible for both representative stations in 6‐SE and the by far lowest numbers in 5‐NE. Due to the definition of t‐events based on the chosen percentile‐based approach, a similar number of t‐event days is observable across all regions and representative stations. Considering ^80^TX in 5‐NE and 6‐SE, t‐events are defined based on generally low or high values in comparison to central European o‐t‐regions 1‐CE, 2‐CN, and 3‐CW. A comparably small number of o‐t‐events is observable in 5‐NE and 6‐SE, even if many o‐event days are present in 6‐SE. This fact underlines the calculated low correlation coefficients between ozone and temperature in 6‐SE presented above.

**Figure 3:**
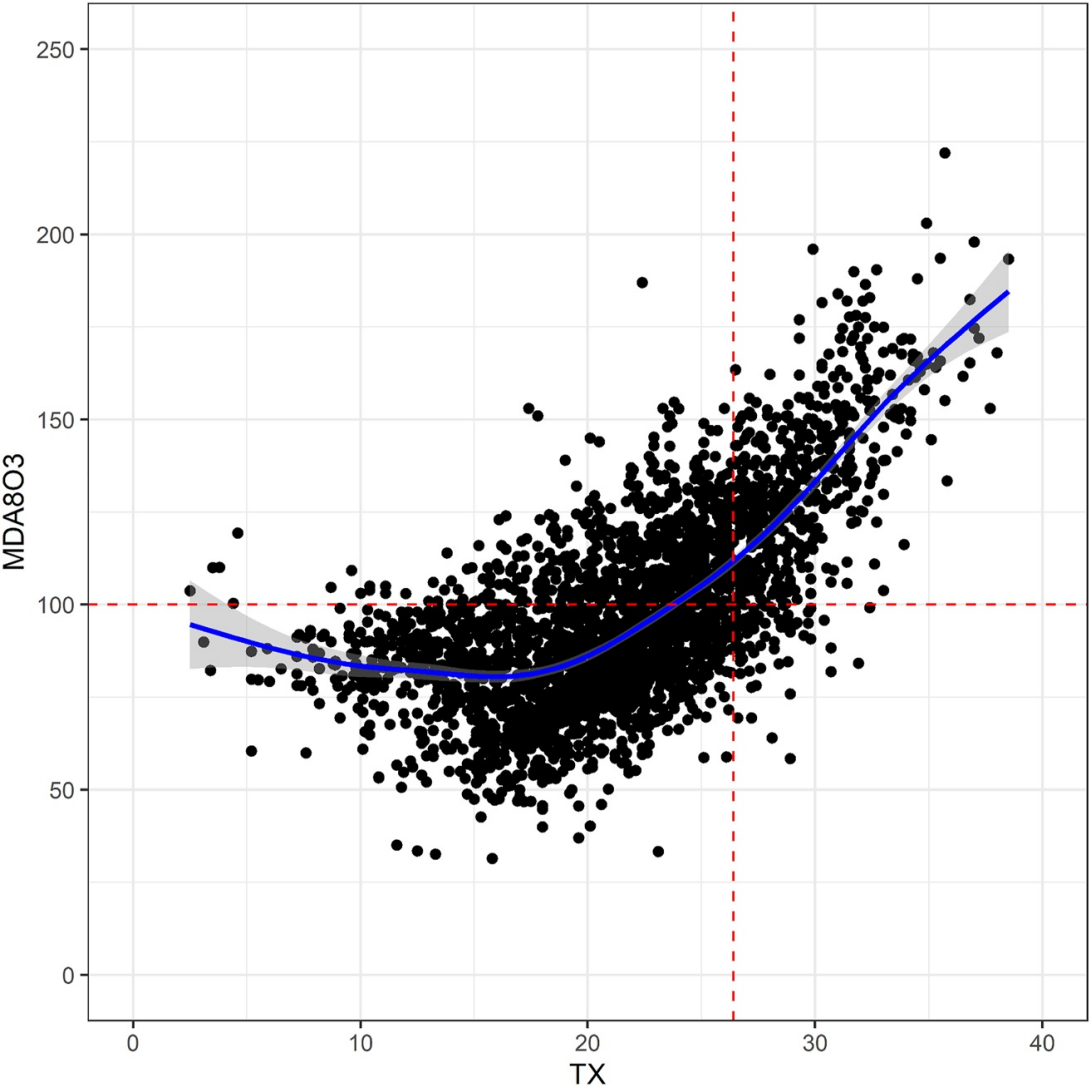
Relationship between MDA8O3 and TX for representative station DEST002 of 1‐CE (Burg in Saxony‐Anhalt). All daily observations from April to September across all years from 2004 to 2018 are considered. A Generalized additive models between both target variables is shown to highlight the linkage between both variables (blue line). Gray shadings illustrate the used confidence interval (0.95). The red horizontal line illustrates the World Health Organisation guideline of 100 μg/m^3^. The red vertical line shows the 80% quantile of all respective observed TX values across all months and years, amounting to 26.4°C.

Generalized additive models (GAM) were applied to analyze the o‐t‐linkage for each o‐t‐region in more depth. Figure 3 shows the o‐t‐relationship exemplarily for the representative suburban background station of 1‐CE, DEST002 (Burg in Saxony‐Anhalt). Please refer to Figure S1 in Supporting Information S1 for all other representative stations. Figure 3 underlines a very direct, approximately linear linkage between both variables as temperature levels approach 20°C. A high proportion of observations lies in the area of a direct response of ozone with clear increases of MDA8O3 at higher TX values. The direct linkage associated with a strong positive ozone response on temperature is also observable as TX levels approach 20°C or 10°C for representative stations of 1‐CE, 2‐CN, and 3‐CW and for the high‐altitude station DESN053 (1214 m) of 4‐HA, respectively.

While comparable results become evident for all four central European regions, deviant relationships are observable for the northern and southern representative stations. The majority of observations in 5‐NE are in lower temperature ranges with 80% quantiles not reaching 20°C. But even for these stations, the upper 20% of TX values already indicate a positive o‐t‐linkage. This observation suggests that similar relationships above 20°C are present at higher air temperature levels. For 6‐SE, it becomes apparent that TX values are by far higher than for all other representative stations. Also, 20% and 80% quantiles surpass 25°C and 33°C as well as 22°C and 30°C for representative stations ES1215 A and ES1666 A, respectively. Thus, o‐t‐events are associated with higher temperatures. The distinct direct linkage with increases of ozone levels due to rising air temperatures is not generally observed for the majority of observations in 6‐SE.

Please note that direct is used in this context throughout the paper to describe the strong relationship between MDA8O3 and TX, relating to a large positive response of MDA8O3 on rising TX levels. This has to be seen independent of the various different direct and indirect causalities determining the o‐t‐relationship. Just to name one of these direct and indirect causalities, respectively: ozone is formed from a complex series of reactions accelerated by warm temperatures (direct); the sunlight‐driven photochemical production of ozone influences the o‐t‐relationship as high solar radiation induces usually also warm air temperatures (indirect).

### 5.2 Statistical Modeling and Climate Change Projection Results

Statistical downscaling models were generated based on the identified station‐specific predictor sets. Similarities and differences in main drivers of compound o‐t‐events across all o‐t‐regions were analyzed. Statistical downscaling models were subsequently applied with ESM data to project frequency shifts of o‐t‐events under two scenario assumptions.

#### 5.2.1 Models and Main Drivers

LR statistical downscaling models were generated based on all selected and preprocessed meteorological and synoptic predictors. Given the geographical proximity of both representative stations of 6‐SE (ES1215 A and ES1666 A), the nine grid‐cell means to generate station‐based meteorological predictor time series are identical. Considering the synoptic predictor, the SOM result showing the smallest topographic product of −0.0374 was selected. The final nine WT, entering the modeling process as numerical codes from one to nine, are shown in Figure S2 in Supporting Information S1. As the array of nodes self‐organizes into a pattern with more similar nodes being in closer proximity and more dissimilar nodes further away, simple clusters can be detected. Please refer to Table S6 in Supporting Information S1 for a more detailed evaluation of the generated WT and an in depth‐analysis of their association with o‐, t‐, and o‐t‐events in the base period.

Table 2 gives an overview of the final LR statistical downscaling model results. Across all stations, a similar, balanced performance considering P and R is achieved. Model fit and performance are in general high for stations in the central European regions. The F1‐Score of the LR models for all but one central European station (ES1599 A) amounts to at least 0.65 (DEHH047) with a maximum of 0.72 (DEST066). A station‐specific maximum F1‐Score of 0.41 is observable across all stations of 5‐NE and 6‐SE. Hence, the LR models in central European regions 1‐CE, 2‐CN, 3‐CW, and 4‐HA clearly outperform the models of 5‐NE and 6‐SE in capturing o‐t‐events.

**Table 2:** Overview of the Logistic Regression Station Model Results

		Regression coefficient								
Station	Ratio	GH	MT	SH	SSRD	WT	P	R	F1‐Score	MF‐R^2^
DEST066	102.60	−0.44	4.29^1^	−1.66^2^	0.76^3^	0.61	0.72	0.72	0.72	0.58
DEBB066	98.60	−0.64	3.84^1^	−1.37^2^	0.82^3^	0.66	0.70	0.70	0.69	0.55
DEST002	95.80	−0.53	4.00^1^	−1.35^2^	0.80^3^	0.63	0.70	0.70	0.69	0.57
NL00807	83.80	−0.67	2.74^1^	−0.48	1.46^2^	0.84^3^	0.68	0.68	0.68	0.56
DEHH047	82.20	−0.41	2.68^1^	−0.51	1.27^2^	0.75^3^	0.65	0.64	0.64	0.53
DEHB002	77.00	−0.76	3.05^1^	−0.59	1.84^2^	0.82^3^	0.69	0.68	0.68	0.61
FR26010	94.00	−0.28	3.29^1^	−1.24^2^	0.48	0.52^3^	0.65	0.66	0.66	0.51
FR07004	88.20	−0.26	3.46^1^	−1.23^2^	0.39^3^	0.16	0.68	0.69	0.68	0.54
ES1599 A	68.40	−0.64^3^	2.25^1^	−0.87^2^	0.39	0.18	0.46	0.44	0.45	0.36
DESN053	129.75	−0.21	4.63^1^	−1.93^2^	‐	0.28^3^	0.70	0.70	0.70	0.54
GB0033 R	16.00	−0.28	1.57^1^	−0.63^3^	0.77^2^	0.50	0.26	0.26	0.29	0.32
GB0567 A	9.20	−1.09^3^	2.18^1^	−0.25	1.84^2^	0.95	0.28	0.38	0.41	0.54
IE0001 R	23.20	−0.22	0.99^2^	−0.33	1.15^1^	0.74^3^	0.26	0.32	0.31	0.28
ES1666 A	55.20	0.39	0.78^1^	−0.50^3^	0.61^2^	−0.17	0.34	0.32	0.32	0.20
ES1215 A	58.20	0.60^2^	0.69^1^	−0.44^3^	0.43	−0.07	0.27	0.27	0.27	0.17

*Note*. The final statistical downscaling model results for all representative stations are shown. Standardized regression coefficients for each predictor variable (GH = geopotential heights, SH = specific humidity, MT = mean air temperatures, SSRD = surface solar radiation downward) are shown. Station‐specific main drivers are highlighted in bold and specified by superscript (MID = 1, SMID = 2, TMID = 3). Model fit and performance are given by Precision (P), Recall (R), F1‐Score, and McFadden's R^2^ (MF‐R^2^). The ratio based on the number of events with respect to the number of predictors is shown as well. Stations are ordered by region (from 1‐CE to 6‐SE) and are framed, respectively, in the table by dashed lines. Within a region, stations are ordered from the first to the last representative station based on the Euclidean distances to their region centroid.

For all stations but one (DESN053), all predictors are significant and included in the model. The standardized regression coefficients as well as the identification of main drivers revealed no clear station‐type‐related differences. Thus, main drivers of o‐t‐events seem to be primary related to larger scale regional influences rather than individual station characteristics. In general, the identified MID and SMID represent by far the most influencing factors of o‐t‐events, especially in central Europe. MT mainly governs o‐t‐events as MID with varying magnitudes. SH and SSRD represent a further influential factor for the vast majority of locations with low humidity and high solar radiation levels favoring the occurrence of o‐t‐events. More sporadically, for some stations, WT and GH are selected as SMID and TMID.

For DESN053 of 4‐HA, SSRD is not a significant predictor and was not kept in the final station model. This might indicate that 4‐HA stations are not as strongly dependent on the local, photochemical in‐situ formation of ozone, but are stronger influenced by, for example, stratospheric O_3_ exchange processes. Nevertheless, DESN053 results show high model fit and performance values with an F1‐Score above 0.7. This as well as the high regression coefficients of other predictors indicate that the selected meteorological and synoptic drivers generally influence compound o‐t‐events at this high‐altitude station.

#### 5.2.2 Climate Change Projections

Climate change projections were generated for all representative stations of central European o‐t‐regions 1‐CE, 2‐CN, 3‐CW, and 4‐HA. Table 3 provides an overview of projected ensemble mean changes statistically downscaled under SSP245 and SSP370 scenario assumptions. Projected frequency shifts range from an increase of about 35%–66% (midcentury) and 56%–110% (late century) for SSP245 and from 48% to 103% (midcentury) and 111%–221% (late century) for SSP370, respectively. Stations DEST066 (1‐CE) and ES1599 A (3‐CW) always show the lowest and highest values, respectively. On average, a growing number of compound event days in central Europe with midcentury and late 21st century projected frequency changes being stronger for SSP370 in comparison to SSP245 becomes apparent. Stronger frequency shifts are assessed for corresponding late century climatic conditions. By considering spatial patterns of projected change, a general gradient from 1‐CE, over 2‐CN, to 3‐CW can be observed across all scenario assumptions and time slice differences. For station DESN053, similar responses comparable to surrounding, low‐altitude stations of 1‐CE become apparent. Respective median, minimum, and maximum projected changes can be found in Table S7 in Supporting Information S1.

**Table 3:** Projected Midcentury and Late 21st Century Ensemble Mean Changes (%)

	SSP245		SSP370	
Station	2041–2060	2081–2100	2041–260	2081–2100
DEST066	34.72	56.32	47.60	111.41
DEBB066	38.87	59.42	49.40	118.40
DEST002	40.08	65.76	56.89	130.31
NL00807	52.01	75.88	62.66	139.92
DEHH047	50.52	74.02	61.87	138.07
DEHB002	54.73	79.99	68.46	152.66
FR26010	51.45	79.02	65.77	147.89
FR07004	55.20	87.06	76.56	177.67
ES1599 A	65.59	110.45	102.93	221.31
DESN053	36.17	61.63	54.98	124.89

*Note*. Climate change projection results for the representative station of all central European o‐t‐regions 1‐CE to 4‐HA, separated by dashed lines, are shown. The ensemble results are based on the number of days with o‐t‐events between the periods 2041–2060 (midcentury) as well as 2081–2100 (late century) compared to the historical Earth System Models (ESM) period 1995–2014 under SSP245 and SSP370 scenario assumptions. The climate change signals of all selected individual ESM showing the same sign, but a different magnitude of change, are considered. Hence, numbers refer to the multi‐model mean across all eight ESM. Stations are listed in accordance with Table 2.

An overall assumption of stationarity of governing statistical and causal relations regarding the predictors themselves as well as between predictors and predictand forms the basis of the applied modeling and climate change projection framework. This includes that all ozone chemistry, transport, and formation conditions and o‐t‐characteristics retain their main characteristics throughout the 21st century. To examine if these basic assumptions are met, projected multi‐model mean midcentury and late century warmings under SSP245 and SSP370 scenario assumptions were analyzed. Region‐specific MT anomalies per scenario and time window, presented in the Table S8 in Supporting Information S1, were hence considered. Projected increases of MT levels do not in general surpass 2.7°C for SSP245 (both midcentury and late century) and SSSP370 (only midcentury conditions) in any central European o‐t‐region. So strong deviant statistical relationships leading to a break of the observed relationships in the modeling periods are rather unlikely under these assumptions. But for late century SSP370 scenario assumptions, in some regions, late century MT anomalies amount to over 4°C.

## 6 Discussion

### 6.1 Regionalization

A regionalization of the European domain based on a hierarchical clustering approach considering daily surface maximum ozone concentrations and air temperature levels from April to September is presented. The result reflects a consistent and robust geographical clustering. O‐t‐regions consist of stations showing comparable spatiotemporal ozone and temperature variabilities, patterns, and characteristics. Region‐specific dynamics seem to have a stronger impact than local site conditions and station types. This includes that station types did not highly influence the assignment of a station to an o‐t‐region. In accordance, the proximity of a station to its centroid, based on Euclidean distances, could not be connected to site characteristics. The observed station‐based patterns generally fit the overall region‐specific conditions of both target variables. This is also particularly true for the representative stations of varying station types identified for all six o‐t‐regions. The results give evidence that interregional dissimilarities are strong in comparison to intraregional variability and differences, including that station types do not substantially affect o‐t‐relationships in comparison to region‐wide influences. This hints to the fact that o‐t‐events in Europe are phenomena of a broader regional scale influenced by larger scale processes. Thus, a region‐specific approach to model and project compound events can uncover widespread, regional variations. The results also highlight the usefulness of synoptic WT in modeling and climate change projection studies.

### 6.2 Present‐Day Relationships and Models

#### 6.2.1 Central Europe

For central European o‐t‐regions 1‐CE, 2‐CN, 3‐CW and to a lesser extent, also for 4‐HA, a strong positive correlation and direct relationship between MDA8O3 and TX in the o‐t‐season from April to September is confirmed. Statistical downscaling models based on logistic regression to assess the relationship between four selected meteorological predictors, synoptic WT, and o‐t‐events verify the physical processes behind ground‐level ozone formation as well as elevated temperature levels. The validity of the models is emphasized as not only model fit and performance values, but also the magnitude of the standardized regression coefficients hint to a strong relationship between the selected predictors and the occurrence of o‐t‐events. The results point to the circumstances that central Europe temperature conditions support a direct o‐t‐linkage with a large portion of observations lying in ranges with linear MDA8O3 responses to rising air temperatures. A clear relationship between compound o‐t‐events and meteorological and synoptic mechanisms is observed for all central European regions. In general, mean air temperatures, specific humidity levels (both at 850 hPa levels) as well as downward‐directed surface solar radiation can be regarded as powerful predictors to assess compound o‐t‐events.

Even though the presented results indicate a substantial relationship between compound o‐t‐events and the identified main drivers also for stations in high‐altitude o‐t‐region 4‐HA, station AT0SON1 shows a lesser o‐t‐linkage due to its unique site conditions. Therefore, results should only be transferred to all other stations of 4‐HA. In future work with differing framework conditions (e.g., by considering variable time periods as base period), further stations being located at similar altitude levels as AT0SON1 may be included in the database and lead to a separation of this station from the given cluster 4‐HA. This will probably result in the formation of a new, separate o‐t‐region, consisting of primarily stations monitoring background, free troposphere ozone values.

#### 6.2.2 Northern and Southern Europe

For 5‐NE and 6‐SE, a deviant picture emerged. Presented results point to the fact that existing temperature and environmental conditions determine a comparably weak o‐t‐linkage and correlation. An absent or low direct dependence of MDA8O3 on temperature levels is reasonable for 5‐NE as the majority of TX observations lay under 20°C. In contrast, high TX values determine o‐t‐events at both representative stations in 6‐SE. A break of the direct o‐t‐relationship with plateauing or even decreasing ozone concentrations under very high air temperatures might be in general anticipated (e.g., Steiner et al., 2010 above 37°C). However, as most of the observations in 6‐SE still lay inside moderate temperature ranges, the weak observed linkage and correlation between TX and MDA8O3 cannot be substantially attributed to this relation. The general presumption of a strong direct relationship of local ozone and air temperature conditions is further based on the assumption of a high proportion of O_3_ in‐situ formation by photochemical reactions. If long‐range transport processes of ozone from various locations outside the specific o‐t‐region strongly determine pollution levels, as for example, Hertig et al. (2020) found for Portugal, a weaker connection of station‐based MDA8O3 levels on local air temperatures can be anticipated. Similar influences and further non‐in‐situ formation factors might be present in 6‐SE with stations spread across Portugal and mainly southern parts of Spain and France. This is further underlined by the observed, comparable low number of co‐occurring o‐t‐events in 6‐SE although a high number of o‐event days are present (in contrast to 5‐NE showing in general low o‐ and o‐t‐event occurrences).

All central European station models clearly outperform the ones of 5‐NE and 6‐SE. The deviant o‐t‐characteristics and linkages as well as the generally observed lower number of o‐t‐events in 5‐NE and 6‐SE may determine the comparably weak predictive power of all southern and northern station models. This might also rely on a general relatively weak role of direct physical predictors on combined elevated ozone concentration and temperature levels in northern and southern Europe. This is also in accordance with the results of previous studies, for example, Otero et al. (2016) who found that preceding conditions based for example, on precursor emissions rather than meteorological variables impact ozone concentrations in these parts of Europe. Furthermore, daily exceedances based on ^80^TX values in 6‐SE already represent comparably high levels (amounting to rounded median values of 30°C and 33°C). Hence, the modeled o‐t‐events are in air temperature ranges approaching the environmental conditions for which a weaker response of MDA8O3 concentrations on TX values can already be expected.

### 6.3 Climate Change Projections

Using the output of eight ESM of the CMIP6 project, the LR models together with ESM data were used for climate change projections. 5‐NE and 6‐SE did not show a strong direct linkage between MDA8O3 and TX levels as well as between selected physical drivers and o‐t‐events, visible by low model fit and performance values. Hence, both o‐t‐regions were discarded for climate change projections. Multi‐model mean changes point in general to an increase in the occurrence of o‐t‐events until the end of the 21st century. This is in accordance with the statistical downscaling modeling results across all central European o‐t‐regions. MT, SH, and SSRD represent in general the most important drivers of o‐t‐events over central Europe. Consequently, projected increases of future air temperatures, solar radiation levels, and related specific humidity changes impact future mean O_3_ concentrations and temperature levels. Hence, a growth in future compound o‐t‐event occurrences under global warming can be expected. Clear interregional differences, but no obvious dependences of the projections on site‐specific characteristics, and hence station types become evident. Assuming stationarity of recent ozone chemistry conditions and underlying processes, this indicates that overall region‐specific environmental changes lead to a similar response to the climate change signals across all stations. For late century SSP370 scenario assumptions, a break of the observed relationships cannot be ruled out for some o‐t‐regions. For 3‐CW, statistically modeled relationships of the base period could not simply be assumed to hold in a projected, warmer central European climate. So, respective climate change projections need to be interpreted with caution. Even if a substantial frequency shift is projected for all central European o‐t‐regions, some areas (e.g., in 2‐CN) that already show nowadays comparably high levels of both variables may be more affected by future changes and should be emphasized in future European‐wide mitigation and adaption strategies.

## 7 Limitations

Some of the results might be influenced by limitations from the study design. The study relies on a strong and direct linkage between MDA8O3 and TX levels as well as on stationarity of all statistical relationships and characteristics. This includes, for example, all ozone chemistry influencing processes and conditions not directly incorporated in the statistical downscaling models. A major source of uncertainty is thus based on the assumption that present‐day o‐t‐relationships are expected to hold across all central European o‐t‐regions under future warming. This fact was accounted for by only considering central European o‐t‐regions for climate change projections and by the evaluation of MT anomalies based on all scenarios and time slice differences. But, nevertheless, this approach relies on the postulation that precursor emissions are anticipated to behave accordingly, which must be considered as a major assumption of the given work. The aim of the study was to analyze o‐t‐events considering future climatic changes, including, but not limited to, temperature. Precursor emissions and their possible future reductions, for example, with regard to European climate neutrality goals, but also further factors like future land use and vegetational changes, may influence the governing present‐day relationships in magnitude and characteristic, but were not directly included in this work. However, a break of the observed and modeled statistical associations of ozone, temperature, meteorology, and synoptic conditions can also not simply be anticipated as a variety of influences, such as for instance precursors emissions transported from extracontinental sources to the identified European o‐t‐regions, might determine future European o‐t‐event occurrences. Station and site characteristics might also influence future changes in o‐t‐events and vary not only in an inter‐ but also intraregional perspective. This fact was mitigated by two primary decisions. At first, only background stations installed to monitor regional background levels representative of the average exposure of the general population rather than locally limited and site‐specific pollution levels like major roads or industrial areas were selected. Second, several representative stations per region based on deviant station characteristics were selected. This selection also enables to include varying site characteristics with alternating pollution regimes and influences in the model and projection process. As in general more NO_x_‐sensitive regimes in rural and VOC‐sensitive regimes in urbanized areas can be expected, the results of the regionalization, modeling, and projection process show primary regional, not station‐type dependencies, and so underline the minor role of regimes in this context. Nevertheless, individual sites of a region might still show strongly deviant developments based on specific local environmental conditions, and a selected station may not be representative for the general exposure of a region anymore. This cannot be accounted for by the chosen approach. The performance of the generated LR models was in general good for central Europe, but individual station model fit, and performance values have always to be kept in mind when interpreting station‐specific developments and climate change projections.

Limitations based on the different resolution of the ESM and ERA5 reanalysis data and thus related interpolation and preprocessing as well as general uncertainties of the CMIP6 climate change projections themselves might exist. ESM data were thus bias‐corrected and the significant distributional differences between the climate model and reanalysis data were evaluated. Nevertheless, the general coarse resolution of some ESMs used for 21st century projections must be pointed out.

Most importantly, it becomes apparent that the applied study framework is to a large extent only suitable for all central European stations. Consequently, for northern and southern parts of Europe, a revised and adapted approach needs to be developed in future health‐relevant o‐t‐event‐studies. Frequency shifts of o‐t events until the end of the 21st century based on adjusted northern and southern European region‐specific, station‐based statistical models, and related projections could subsequently be compared with the here presented central European results and possible relevant differences assessed.

## 8 Conclusions

Six ozone and temperature regions together with 15 representative stations with different site characteristics and air quality settings were identified. A region‐specific analysis of both target variables, including the evaluation of the relationship between meteorological and synoptic conditions and compound o‐t‐events, was conducted. A strong and direct relationship and correlation between ozone and temperature as well as a good performance of the statistical downscaling models were primarily found in all central European o‐t‐regions. Consequently, climate change projections were only generated for central Europe. Sharp increases of o‐t‐events, considering 20‐year time slice differences under SSP245 and SSP370 scenario assumptions, were in general assessed resulting in a considerable intensified health burden for the central European population until the end of the 21st century.

Concluding, compound ozone and temperature events combining two natural hazards and composing a substantial health risk throughout different regions of central Europe should be the focus of further studies, but also upcoming European climate change mitigation and adaption strategies. An adjusted and adapted approach focusing on northern and southern parts of Europe should be incorporated in future work of health‐relevant o‐t‐event‐studies.

## Conflict of Interest

The authors declare that they have no conflicts of interest.

## Supporting information

Supporting Information S1

## Data Availability

The authors acknowledge the data providers of the ECA&D and Air Quality eReporting data sets. Respective ECA&D data (Klein Tank et al., 2002) used in this study are available at the following website: https://www.ecad.eu/dailydata/predefinedseries.php (accessed 3 July 2020). Air Quality eReporting data (EEA, 2017) are available at https://aqportal.discomap.eea.europa.eu/with the raw data accessible via and downloaded on 3 July 2020 at https://discomap.eea.europa.eu/map/fme/AirQualityExport.htm (since 2013) and https://discomap.eea.europa.eu/map/fme/AirQualityExportAirBase.htm (2004–2012). The authors also acknowledge the European Centre for Medium‐Range Weather Forecasts for provision of the ERA5 data set as well as the World Climate Research Programme's Working Group on Coupled Modelling, which is responsible for CMIP, and we thank the climate modeling groups for producing and making available their model output. ERA5 (Hersbach & Dee, 2016) and CMIP6 (Eyring et al., 2016) data are available at https://cds.climate.copernicus.eu/and https://esgf-node.llnl.gov/projects/cmip6/, respectively. Most of the data preparation and analysis including regionalization, model building, and projections were conducted using the Python programming language (version 3.7.4, IDE PyCharm).
