# Supplementary material for: Using Clustering, Statistical Modeling, and Climate Change Projections to Analyze Recent and Future Region‐Specific Compound Ozone and Temperature Burden Over Europe
Source: Geohealth. 2022 Apr 16;6(4):e2021GH000561. doi: 10.1029/2021GH000561 (PMC9012997; doi:10.1029/2021GH000561)

GeoHealth

Supporting Information for

Using clustering, statistical modeling, and climate change projections to analyze recent and future region-specific compound ozone and temperature burden over Europe

Sally Jahn[1], Elke Hertig[2]

[1]Regional Climate Change and Health, Institute of Geography and Faculty of Medicine,

University of Augsburg, Werner-von-Siemens-Straße 6, 86159 Augsburg, Germany

[2]Regional Climate Change and Health, Faculty of Medicine,

University of Augsburg, Werner-von-Siemens-Straße 6, 86159 Augsburg, Germany

Contents of this file

Tables S1 to S8
Figures S1 to S2

Table S1 *Abbreviations and Acronyms*

General	Explanation
MDA8O3	Daily maximum ozone values based on 8-hour running means
NO_x	Nitrogen oxides
O_3	Tropospheric, ground-level ozone
o- / t- / o-t-(events, season etc.)	Ozone- / temperature- / Ozone - temperature (events, season etc.)
PAN	Peroxyacetyl nitrate
TX; ^{80}TX	Daily maximum surface air temperature; Daily 80th percentiles based on a 31-day window, 2004-2018
VOCs	Volatile organic compounds
Ward's method	Ward variance minimization algorithm
WHO	World Health Organization

Predictors	Explanation
GH	Geopotential heights at 850 hPa
MID	Most important driver
MT	Mean air temperatures at 850 hPa
SH	Specific humidity at 850 hPa
SMID	Second most important driver
SSRD	Surface solar radiation downwards
TMID	Third most important driver
WT	Weather type(s)

O-t-Regions	Explanation
1-CE	Central Eastern
2-CN	Central Northern
3-CW	Central Western
4-HA	Central High Altitude
5-NE	Northern European
6-SE	Southern European

Models/Projections	Explanation
CMIP5/6	Coupled Model Intercomparison Project Phase 5/6
ESM	Earth System Model(s)
F1-Score	Metric represents the harmonic mean and maintains a balance between R and P.
GAM	Generalized additive model(s)
LR	Logistic Regression
MF-R^2	McFadden's R^2
P	Precision<br>Metric is used to measure the fraction of the predicted events that did actually occur.
R	Recall<br>Metric is used to measure the fraction of actual, observed events that are correctly predicted.
SMOTE	Synthetic Minority Oversampling Technique

Table S2 *Overview Station Pairs*

No.	O_3 station code	O_3 station name	O_3 station type	O_3 station longitude [°]	O_3 station latitude [°]	O_3 station altitude [m a.s.l.]	TX station code	TX station name	Distance [km]	Altitude difference [m]	O-t-region
1	AT0SON 1	Sonnblick	1	12.96	47.05	3106	15	SONNBLICK	0.81	0	4-HA
2	AT4S156	Braunau Zentrum, Kolpingplatz	3	13.04	48.26	350	4647	SIMBACH/INN	1.81	-10	1-CE
3	AT51200	Salzburg Lehener Park, Franz-Martin-Straße 1	2	13.03	47.82	455	14	SALZBURG	3.01	18	1-CE
4	AT60138	Graz Nord Gösting	3	15.41	47.10	348	12	GRAZ	3.05	-18	1-CE
5	AT72113	Innsbruck Sadrach	3	11.37	47.27	670	13	INNSBRUCK	2.57	93	1-CE
6	AT90LA A	Wien Laaer Berg	2	16.39	48.16	250	16	WIEN	8.58	52	1-CE
7	BETR001	41R001 - MOLENBEEK	2	4.33	50.85	20	17	UCCLE	5.98	-80	2-CN
8	DEBB021	Potsdam-Zentrum	2	13.06	52.40	31	54	POTSDAM	2.02	-50	1-CE
9	DEBB048	Neuruppin	3	12.81	52.93	43	475	NEURUPPIN	2.96	5	1-CE
10	DEBB053	Hasenholz	1	14.02	52.56	88	4430	MUNCHEBERG	8.95	25	1-CE
11	DEBB064	Cottbus	2	14.33	51.75	75	4014	COTTBUS	3.59	6	1-CE
12	DEBB065	Lütte (Belzig)	1	12.56	52.19	111	4766	WIESENBURG	10.61	-76	1-CE
**13**	**DEBB066**	**Spreewald**	**1**	**14.06**	**51.90**	**52**	**4370**	**LUBBEN-BLUMENFELDE**	**12.48**	**-5**	**1-CE**
14	DEBB067	Nauen	3	12.89	52.61	31	4808	BERGE	6.64	-9	1-CE

15	DEBE010	Berlin Wedding	2	13.35	52.54	35	4563	BERLIN-MITTE	2.54	0	1-CE
16	DEBE027	Berlin Marienfelde	1	13.37	52.40	45	4556	BERLIN-LICHTENRADE	3.30	-2	1-CE
17	DEBE032	Berlin Grunewald (3.5 m)	1	13.23	52.47	50	4588	BERLIN-ZEHLENDORF	4.81	5	1-CE
18	DEBE051	Berlin Buch	3	13.48	52.64	60	4529	BERLIN-BUCH	1.91	0	1-CE
19	DEBW023	Weil am Rhein	3	7.63	47.59	275	4042	EIMELDINGEN	5.35	11	3-CW
20	DEBW024	Ludwigsburg	3	9.17	48.90	302	3506	STUTTGART-SCHNARRENBERG	8.06	-12	3-CW
21	DEBW027	Reutlingen	2	9.21	48.49	385	4554	REUTLINGEN-BETZINGEN	2.54	25	3-CW
22	DEBW039	Villingen-Schwenningen	3	8.46	48.05	700	4712	VILLINGEN-SCHWENNINGEN	0.35	-20	3-CW
23	DEBW042	Bernhausen	3	9.23	48.68	370	2763	STUTTGART/ECHTERDINGEN	1.25	-1	3-CW
24	DEBW052	Konstanz	2	9.17	47.66	403	495	KONSTANZ	2.22	-40	3-CW
25	DEBW073	Neuenburg	3	7.57	47.82	223	4286	MULLHEIM	5.49	-50	3-CW
26	DEBW076	Baden-Baden	3	8.22	48.77	148	4284	BADEN-BADEN-GEROLDSAU	5.40	-92	3-CW
27	DEBW081	Karlsruhe-Nordwest	2	8.36	49.03	114	51	KARLSRUHE	1.36	2	3-CW
28	DEBW084	Freiburg	2	7.83	48.00	262	4115	FREIBURG IM BREISGAU-HERDERN	2.46	7	3-CW

29	DEHB001	Bremen-Mitte	2	8.82	53.08	10	4884	BREMEN-SEEFAHRTSCHULE	3.45	6	2-CN
**30**	**DEHB00 2**	**Bremen-Ost**	**2**	**8.92**	**53.06**	**7**	**42**	**BREMEN**	**8.02**	**3**	**2-CN**
31	DEHB005	Bremerhaven-Hansastraße	2	8.57	53.56	3	4885	BREMERHAVEN	3.16	-4	2-CN
32	DEHH008	Hamburg Sternschanze	2	9.97	53.56	15	4180	HAMBURG-BOTANISCHER GARTEN	1.39	1	2-CN
33	DEHH033	Hamburg Flughafen Nord	2	10.00	53.64	13	47	HAMBURG FUHLSBUETTEL	0.64	2	2-CN
**34**	**DEHH04 7**	**Hamburg Bramfeld**	**3**	**10.11**	**53.63**	**31**	**4186**	**HAMBURG-WANDSBEK**	**5.08**	**13**	**2-CN**
35	DEHH050	Hamburg Neugraben	3	9.86	53.48	3	4182	HAMBURG-NEUWIEDENTHAL	2.73	0	2-CN
36	DEMV00 7	Rostock-Stuthof	3	12.17	54.16	5	472	ROSTOCK-WARNEMUNDE	6.40	1	2-CN
37	DENW03 8	Mülheim-Styrum	2	6.87	51.45	39	4074	ESSEN-BREDENEY	8.93	-111	2-CN
38	DENW04 2	Krefeld-Linn	2	6.64	51.34	36	4030	DUISBURG-FRIEMERSHEIM	7.75	5	2-CN
39	DENW05 9	Köln-Rodenkirchen	3	6.99	50.89	50	4298	KOLN-BOTANISCHER GARTEN	8.16	5	2-CN
40	DENW06 5	Netphen Rothaargebirge	1	8.19	50.93	641	4514	BAD-STUNZEL BERLEBURG	13.81	31	4-HA
41	DENW07 1	Düsseldorf-Lörick	2	6.73	51.25	38	4029	DUSSELDORF-SUDFRIEDHOF	5.67	1	2-CN

42	DENW078	Ratingen-Tiefenbroich	3	6.82	51.30	42	479	DUSSELDORF	3.54	5	2.CN
43	DENW079	Leverkusen-Manfort	3	7.00	51.03	50	4351	KOLN-STAMMHEIM	4.66	7	2-CN
44	DENW080	Solingen-Wald	3	7.05	51.18	208	4651	SOLINGEN-HOHENSCHEID	5.59	54	2-CN
45	DENW081	Borken-Gemen	1	6.87	51.86	44	4841	BORKEN IN WESTFALEN	1.59	-4	2-CN
46	DENW096	Mönchengladbach-Rheydt	3	6.43	51.15	83	4423	MONCHENGLADBACH	5.14	34	2-CN
47	DESH001	Altendeich	1	9.59	53.67	8	4195	HASELDORF	4.09	5	2-CN
48	DESH008	Bornhöved	1	10.24	54.09	45	4578	RUHWINKEL	1.14	9	2-CN
49	DESH023	Lübeck-St. Jürgen	2	10.70	53.84	12	4372	LUBECK-BLANKENSEE	2.86	7	2-CN
50	DESL002	Bexbach Schule	3	7.26	49.36	275	4458	NEUNKIRCHEN-WELLESWEILER	3.10	39	3-CW
51	DESL011	Saarbrücken-Eschberg	2	7.04	49.24	315	4584	SAARBRUCKEN-SANKT JOHANN	1.99	122	3-CW
52	DESN004	Bautzen	2	14.44	51.18	203	4384	KR. BAUTZEN KUBSCHUTZ	4.58	-29	1-CE
53	DESN049	Carlsfeld	1	12.61	50.43	896	4950	CARLSFELD	0.14	-1	4-HA
54	DESN051	Radebeul-Wahnsdorf	1	13.68	51.12	246	43	DRESDEN WAHNSDORF	0.65	0	1-CE
55	DESN052	Zinnwald	1	13.75	50.73	877	4801	ZINNWALD-GEORGENFELD	0.18	0	4-HA
**56**	**DESN053**	**Fichtelberg**	**1**	**12.95**	**50.43**	**1214**	**488**	**FICHTELBERG**	**0.15**	**1**	**4-HA**

57	DESN059	Leipzig-West	2	12.30	51.32	115	4344	LEIPZIG-HOLZHAUSEN	10.44	-23	1-CE
58	DESN080	Schkeuditz	1	12.23	51.40	122	482	LEIPZIG-SCHKEUDITZ	4.45	-9	1-CE
59	DESN081	Plauen-DWD	3	12.13	50.48	385	4522	PLAUEN	0.17	-1	1-CE
**60**	**DEST002**	**Burg**	**3**	**11.86**	**52.27**	**47**	**4679**	**THEESSEN**	**13.21**	**-14**	**1-CE**
61	DEST011	Wernigerode/Bahnhof	2	10.79	51.84	235	4758	WERNIGERODE	1.70	1	1-CE
62	DEST028	Zeitz	2	12.14	51.05	156	4797	ZEITZ	0.21	-14	1-CE
63	DEST039	Brocken	1	10.62	51.80	1130	2006	BROCKEN	0.19	-12	4-HA
**64**	**DEST066**	**Wittenberg/Bahnstrasse**	**2**	**12.66**	**51.87**	**71**	**4776**	**WITTENBERG**	**2.46**	**-34**	**1-CE**
65	DEST077	Magdeburg/West	2	11.61	52.13	58	477	MAGDEBURG	3.33	-18	1-CE
66	DETH009	Gera Friedericistr.	2	12.07	50.88	190	3990	GERA-LEUMNITZ	3.89	-121	1-CE
67	DETH020	Erfurt Krämpferstr.	2	11.04	50.98	195	487	ERFURT-BINDERSLEBEN	5.27	-121	1-CE
68	DETH026	Dreißigacker	1	10.38	50.56	450	486	MEININGEN	0.14	0	1-CE
69	DETH027	Neuhaus	1	11.13	50.50	840	4450	NEUHAUS AM RENNWEG	0.17	-5	4-HA
70	DETH041	Jena Dammstr.	2	11.60	50.93	140	49	JENA STERNWARTE	1.18	-15	1-CE
71	DETH061	Hummelshain	1	11.66	50.79	357	4415	SCHMIERITZ-WELTWITZ	11.15	8	1-CE
72	DEUB028	Zingst	1	12.72	54.44	1	4353	BARTH	10.75	-2	2-CN
73	DEUB029	Schmücke	1	10.77	50.65	937	4617	SCHMUCKE	0.19	0	4-HA

74	DK0045A	"Copenhagen/1259"	2	12.56	55.70	25	116	KOEBENHAVN: LANDBOHOJSKOLE N-1	2.61	16	2-CN
75	EE0018A	Oismäe	2	24.65	59.41	6	11363	TALLINN	3.19	-27	2-CN
76	ES0124A	ARTURO SORIA	2	-3.64	40.44	698	230	MADRID - RETIRO	4.56	31	6-SE
**77**	**ES1215A**	**Amposta (Sant Domènec - Itàlia)**	**3**	**0.58**	**40.71**	**8**	**236**	**TORTOSA - OBSERVATORIO DEL EBRO**	**14.77**	**-36**	**6-SE**
78	ES1421A	TERUEL	2	-1.11	40.34	915	3966	TERUEL	1.68	15	6-SE
79	ES1443A	BURGOS 4	2	-3.64	42.34	929	414	BURGOS-VILLAFRIA	2.21	39	3-CW
80	ES1472A	ITURRAMA	2	-1.65	42.81	449	3950	PAMPLONA (OBSERVATORIO)	1.67	7	3-CW
81	ES1516A	RIO SAN PEDRO	2	-6.23	36.52	1	415	CADIZ	3.73	0	6-SE
82	ES1529A	TETUÁN	2	-3.79	43.47	30	1392	SANTANDER CENTRO	2.34	-34	3-CW
83	ES1535A	ALBACETE	3	-1.85	38.98	686	3906	ALBACETE OBS.	3.13	12	6-SE
84	ES1572A	PURIFICACIÓN TOMÁS	2	-5.87	43.37	276	3913	OVIEDO	2.06	-60	3-CW
**85**	**ES1599A**	**PAGOETA**	**1**	**-2.16**	**43.25**	**225**	**234**	**SAN SEBASTIAN - IGUELDO**	**11.32**	**-26**	**3-CW**
86	ES1602A	LA CIGÜEÑA	2	-2.43	42.46	385	1398	LOGRONO-AGONCILLO	8.09	32	3-CW
87	ES1604A	BELLVER	3	2.62	39.56	117	3918	PALMA DE MALLORCA CMT	1.00	114	6-SE
88	ES1612A	MAJADAHONDA	3	-3.87	40.45	722	3947	MADRID/CUATROVIENTOS	10.17	35	6-SE

89	ES1615A	CÁCERES	2	-6.36	39.47	389	3921	CACERES CIUDAD	1.27	-70	6-SE
90	ES1619A	VALÈNCIA-VIVERS	2	-0.37	39.48	11	237	VALENCIA	0.32	0	6-SE
91	ES1624A	ELX-AGROALIMENTARI	3	-0.68	38.24	44	309	ALICANTE EL ALTET	10.79	1	6-SE
92	ES1638A	BERMEJALES	2	-5.98	37.35	26	423	SEVILLA/SAN PABLO	11.77	-8	6-SE
93	ES1641A	RENOVALES	2	-0.89	41.64	220	238	ZARAGOZA AEROPUERTO	9.95	-27	3-CW
**94**	**ES1666A**	**Tarragona (parc de la Ciutat)**	**2**	**1.24**	**41.12**	**13**	**1401**	**REUS/AEROPUERTO**	**6.35**	**-58**	**6-SE**
95	ES1713A	PARQUE EUROPA	2	-2.90	43.25	76	1393	BILBAO AEROPUERTO	4.81	34	3-CW
96	FI00352	Oulanka	1	29.40	66.32	310	7765	KUUSAMO KIUTAKONGAS	6.33	150	5-NE
97	FI00425	Kallio 2	2	24.95	60.19	18	28	HELSINKI KAISANIEMI	1.39	14	2-CN
98	FR02004	Martigues P. Central	2	5.04	43.42	107	39	MARIGNANE	14.15	98	6-SE
99	FR04149	MONTGERON	2	2.46	48.71	68	11249	ORLY	5.47	-21	3-CW
**100**	**FR07004**	**Montferrand**	**2**	**3.11**	**45.80**	**340**	**750**	**CLERMONT-FERRAND**	**2.98**	**9**	**3-CW**
101	FR08017	Périurbaine Sud	3	3.91	43.57	5	2207	MONTPELLIER-AEROPORT	4.20	3	6-SE
102	FR08712	St Estève	3	2.84	42.72	61	36	PERPIGNAN	3.29	19	6-SE
103	FR09015	Les Couronneries	3	0.36	46.59	119	749	POITIERS - BIARD	3.48	-4	3-CW
104	FR12004	ECOLE M.JACQUIER	2	1.42	43.58	143	33	TOULOUSE-BLAGNAC	5.97	-8	3-CW

105	FR14033	STE SAVINE	2	4.05	48.30	119	11243	TROYES-BARBEREY	3.98	7	3-CW
106	FR20048	SAINT EXUPERY	3	5.07	45.75	217	37	LYON - ST EXUPERY	3.02	-18	3-CW
107	FR21019	IFS Caen sud	2	-0.35	49.15	22	738	CAEN-CARPIQUET	8.13	-45	2-CN
108	FR22014	Spicheren(14)	3	6.96	49.20	340	4850	SAARBRUCKEN-BURBACH	5.40	150	3-CW
109	FR24007	ANTIBES JEAN MOULIN	3	7.09	43.60	81	757	NICE	10.70	79	6-SE
110	FR25040	Mesnil Esnard	3	1.16	49.41	160	2184	ROUEN - BOOS	3.21	9	2-CN
**111**	**FR26010**	**Station DAIX**	**3**	**5.00**	**47.35**	**332**	**745**	**DIJON-LONGVIC**	**10.80**	**113**	**3-CW**
112	FR31001	GRAND PARC	2	-0.58	44.86	3	34	BORDEAUX-MERIGNAC	9.47	-44	3-CW
113	FR33211	ANNEMASSE	2	6.24	46.20	441	241	GENEVE OBSERVATOIRE	6.99	36	3-CW
114	FR34024	Joué lès Tours	2	0.65	47.34	90	2190	TOURS	12.39	-18	3-CW
115	FR34032	Leblanc	2	2.40	47.08	133	32	BOURGES	4.30	-28	3-CW
116	FR35007	PALAIS S/ V.- Garros	3	1.31	45.87	333	2195	LIMOGES - BELLEGARDE	10.51	-69	3-CW
117	GB0002R	Eskdalemuir	1	-3.21	55.32	255	272	ESKDALEMUIR	0.42	13	5-NE
118	GB0006R	Lough Navar	1	-7.90	54.44	130	1822	LOUGH NAVAR FOREST	0.19	4	5-NE
**119**	**GB0033R**	**Bush Estate**	**1**	**-3.21**	**55.86**	**180**	**1831**	**PENICUIK**	**4.45**	**-5**	**5-NE**
120	GB0038R	Lullington Heath	1	0.18	50.79	125	1864	EASTBOURNE	8.14	118	2-CN
121	GB0566A	London Bloomsbury	2	-0.13	51.52	20	1859	HAMPSTEAD	5.64	-117	5-NE

**122**	**GB0567A**	**Belfast Centre**	**2**	**-5.93**	**54.60**	**10**	**1821**	**STORMONT CASTLE**	**6.45**	**-46**	**5-NE**
123	GB0584A	Leeds Centre	2	-1.55	53.80	78	1847	BRADFORD	14.84	-56	5-NE
124	GB0642A	London Hillingdon	2	-0.46	51.50	34	1860	HEATHROW	2.11	9	5-NE
125	GB0643A	Leamington Spa	2	-1.53	52.29	55	1853	WELLESBOURNE	10.39	8	5-NE
126	HU0023A	"Debrecen Kalotaszeg"	2	21.62	47.51	111	852	DEBRECEN AIRPORT	2.77	4	1-CE
127	HU0029A	"Pecs Boszorkany	3	18.21	46.08	200	849	PECS POGANY	8.48	-2	1-CE
**128**	**IE0001R**	**Kerry Valentia Observatory**	**3**	**-10.24**	**51.94**	**10**	**123**	**VALENTIA OBSERVATORY**	**1.30**	**1**	**5-NE**
129	IE0028A	Dublin Rathmines Wynnefield Road	2	-6.28	53.35	25	1704	DUBLIN (GLASNEVIN)	1.93	4	5-NE
130	IE0031R	Galway Mace Head	1	-9.90	54.33	8	2143	BELMULLET	12.41	-1	5-NE
131	IE0090A	Monaghan Kilkitt Waterworks	1	-6.88	54.07	170	968	CARRICKMACROSS (DUNOGE)	12.96	82	5-NE
132	LU0101A	Luxembourg Bonnevoie	2	6.14	49.60	275	203	LUXEMBOURG AIRPORT	5.68	-101	2-CN
133	NL00107	"Posterholt-Vlodropperweg"	1	6.04	51.12	32	4207	HEINSBERG-SCHLEIDEN	9.66	-25	2-CN
134	NL00133	"Wijnandsrade-Opfergeltstraat"	3	5.88	50.90	96	168	MAASTRICHT	8.46	-18	2-CN
135	NL00235	"Huijbergen-Vennekenstraat"	1	4.36	51.43	18	604	WOENSDRECHT	1.92	4	2-CN
136	NL00318	"Philippine-Stelleweg"	1	3.75	51.30	5	2571	WESTDORPE	11.05	4	2-CN
137	NL00404	"Den Haag-Rebecquestraat"	2	4.29	52.08	2	10961	VOORSCHOTEN	12.16	3	2-CN

138	NL00437	"Westmaas-Groeneweg"	1	4.45	51.79	-1	3192	ROTTERDAM-GEULHAVEN	14.99	-4	2-CN
139	NL00722	"Eibergen-Lintveldseweg"	1	6.61	52.09	19	454	HUPSEL	4.43	-10	2-CN
140	NL00738	"Wekerom-Riemterdijk"	1	5.71	52.11	18	2563	DEELEN	12.87	-32	2-CN
**141**	**NL00807**	**"Hellendoorn-Luttenbergerweg"**	**1**	**6.40**	**52.39**	**7**	**453**	**HEINO**	**11.02**	**4**	**2-CN**
142	NL00818	"Barsbeek-De Veenen"	1	6.02	52.65	1	411	MARKNESSE	10.22	4	2-CN
143	NL00918	"Balk-Trophornsterweg"	1	5.57	52.92	1	596	STAVOREN	12.97	2	2-CN
144	NL00934	"Kollumerwaard-Hooge Zuidwal"	1	6.28	53.33	1	413	LAUWERSOOG	10.31	-1	2-CN
145	NO0015R	Tustervatn	1	13.91	65.83	440	18266	VARNTRESK	13.11	34	5-NE
146	PL0004R	IMGW Łeba - Rąbka	3	17.53	54.75	2	332	LEBA	0.09	0	2-CN
147	PL0044A	Warszawa-Podleśna	2	20.96	52.28	98	209	WARSZAWA-OKECIE	13.14	-9	1-CE
148	PT03087	Restelo	2	-9.21	38.70	143	214	LISBOA GEOFISICA	5.39	66	6-SE
149	SE0001A	Malmö Rådhuset	2	13.00	55.61	25	5177	MALMO_2	1.55	22	2-CN
150	SE0004A	Göteborg Femman	2	11.97	57.71	31.3	462	GOTEBORG A	1.61	26.3	2-CN
151	SE0005R	Bredkälen	1	15.32	63.85	380	5696	HALLHAXASEN_A	8.41	5	5-NE
152	SE0013R	Esrange	1	21.06	67.88	524	5859	ESRANGE	1.30	189	5-NE
153	SE0014R	Råö	1	11.91	57.39	10	3512	NIDINGEN A	9.99	8	2-CN
154	SE0022A	Stockholm Torkel Knutssongatan	2	18.06	59.32	58	5464	STOCKHOLM_A	2.57	14	2-CN

155	SE0035R	Vindeln	1	19.77	64.24	271	5714	VINDELN-SUNNANSJONAS	5.42	34	5-NE
156	SE0054A	Lund Spyken	2	13.20	55.70	60	463	LUND	0.11	-13	2-CN
157	SI0003A	Ljubljana Bezigrad	2	14.51	46.07	299	228	LJUBLJANA BEZIGRAD	0.33	0	1-CE
158	SI0008R	Iskrba	1	14.86	45.56	540	3311	KOCEVJE	5.42	73	1-CE
159	SI0033A	Murska Sobota-Rakican	1	16.19	46.65	188	3335	MURSKA SOBOTA-RAKICAN	5.43	0	1-CE
160	SI0034A	Nova Gorica	2	13.65	45.96	113	3326	BILJE	7.39	58	1-CE
161	SK0004R	"Stara Lesna - AU SAV EMEP/O3"	1	20.29	49.15	808	334	POPRAD/TATRY	10.47	114	1-CE

Note. All 161 ozone stations with station code, name, type and specific location metadata. O_3 station types are specified as follows: 1 = rural, 2 = urban, 3= suburban. Station codes and names of the assigned temperature stations are given. Respective distance and altitude difference values for each station pair are depicted. The o-t-region each station pair is assigned to is given as well. Stations are listed in alphabetical order based on the O_3 station code. Representative stations are framed and highlighted in bold.

Table S3 *ERA5-ESM pairs with Significant Distributional Differences*

ESM	Station	O-t-region	Variable
CanESM5	DESN053	4-HA	GH
	ES1599A	3-CW	SH
FGOALS-g3	ES1215A	6-SE	SH
	ES1666A	6-SE	SH
	NL00807	2-CN	GH
INM-CM5-0	ES1215A	6-SE	SH
	ES1599A	3-CW	SH
	ES1666A	6-SE	SH
	FR07004	3-CW	SH
IPSL-CM6A-LR	DEBB066	1-CE	SSRD
	DEST066	1-CE	SSRD
MIROC6	GB0033R	5-NE	MT

Note. 12 ERA5-ESM pairs that show significant distributional differences for a specific meteorological predictor based on the evaluation of monthly time series data by applying a two-sample Kolmogorov-Smirnov test. Distribution differences were tested on the 95% significance level. In general, 4 (predictor variables) x 15 (representative stations) x 8 (ESM) = 480 ERA5-ESM predictor variable pairs were tested. No statistical downscaling projections were generated for 5 of the 12 depicted representative stations, as all stations of 5-NE and 6-SE were discarded from further analysis after the evaluation of o-t-characteristics in the base period and the subsequent modeling process.

Table S4 *Daily Weather Type Occurrences (%)*

ESM	WT1	WT2	WT3	WT4	WT5	WT6	WT7	WT8	WT9
BCC-CSM2-MR	13.09	8.55	13.72	9.59	9.67	9.40	9.97	12.40	13.61
CanESM5	13.36	12.38	9.48	12.95	8.22	9.13	7.84	14.21	12.43
FGOALS-g3	12.65	11.17	10.79	11.53	12.19	9.26	8.72	12.35	11.34
INM-CM5-0	12.27	15.77	10.27	12.35	8.66	10.96	3.72	15.27	10.74
IPSL-CM6A-LR	11.80	12.65	10.93	13.55	9.64	9.95	9.86	13.33	8.28
MIROC6	13.61	14.89	5.82	13.91	9.86	11.12	6.64	15.77	8.39
MPI-ESM1-2-HR	12.54	12.19	10.79	13.50	8.77	10.98	10.38	12.73	8.11
MRI-ESM2-0	12.87	11.45	10.49	12.19	10.55	9.75	10.46	12.62	9.62
**ERA5**	14.32	12.20	9.22	11.99	11.07	9.18	9.76	12.31	9.95

Note. The relative numbers (%) of daily weather type occurrences per ESM as well as for ERA5 reanalysis data are shown. The relative numbers are based on daily WT time series data considering the chosen historical ESM period 1995-2014 for all eight climate models, while reanalysis WT numbers refer to the base period 2004-2018.

Table S5 *Overview Representative Stations*

Station	Position	MDA8O3 mean (min / max)	MDA803 median (25th / 75th)	TX mean (min / max)	TX median (25th / 75th)	^{80}TX mean (min / max)	^{80}TX median (25th / 75th)	Total number of o-/ t- / o-t-event days [-]	Relative number of ozone / temperature / o-t-events [%]
**1-CE - 12 rural, 18 urban and 9 suburban (total: 39) stations**									
DEST066 (urban)	1	100.31 (27.00 / 231.37)	96.51 (81.00 / 116.17)	21.63 (1.80 / 37.80)	21.80 (17.80 / 25.50)	25.58 (16.54 / 29.92)	26.60 (23.10 / 28.00)	1226 / 547 / 513	44.66 / 19.93 / 18.69
DEBB066 (rural)	3	98.68 (21.00 / 195.00)	96.17 (81.69 / 113.80)	22.12 (2.30 / 37.90)	22.40 (18.30 / 25.90)	26.02 (17.60 / 30.40)	26.92 (23.62 / 28.52)	1188 / 540 / 493	43.28 / 19.67 / 17.96
DEST002 (suburban)	9	98.32 (31.40 / 222.01)	94.01 (79.80 / 113.00)	21.63 (2.50 / 38.50)	21.90 (17.90 / 25.30)	25.53 (16.72 / 29.80)	26.50 (23.40 / 27.92)	1105 / 547 / 479	40.26 / 19.93 / 17.45
**2-CN - 16 rural, 20 urban and 11 suburban (total: 47) stations**									
NL00807 (rural)	1	88.08 (11.96 / 242.48)	83.09 (68.88 / 101.71)	20.05 (6.00 / 36.70)	20.10 (16.90 / 23.20)	23.36 (16.20 / 27.00)	24.10 (21.44 / 25.30)	720 / 544 / 419	26.23 / 19.82 / 15.26
DEHH047 (suburban)	4	86.12 (15.00 / 225.05)	83.00 (69.05 / 99.18)	20.02 (3.30 / 37.80)	20.00 (16.50 / 23.40)	23.65 (15.30 / 28.22)	24.40 (21.52 / 25.72)	652 / 548 / 411	23.75 / 19.96 / 14.97
DEHB002 (urban)	5	83.54 (8.44 / 211.02)	80.21 (65.45 / 98.24)	20.32 (3.80 / 36.80)	20.40 (16.90 / 23.80)	23.92 (16.20 / 28.30)	24.70 (21.82 / 25.90)	611 / 538 / 385	22.26 / 19.60 / 14.03
**3-CW - 1 rural, 20 urban and 12 suburban (total: 33) stations**									
FR26010 (suburban)	1	100.30 (32.00 / 204.00)	97.00 (83.00 / 114.50)	22.62 (5.40 / 37.70)	22.70 (19.00 / 26.40)	26.51 (19.00 / 30.80)	27.12 (23.80 / 29.40)	1204 / 538 / 470	43.86 / 19.60 / 17.12
FR07004 (urban)	2	98.18 (24.00 / 185.00)	96.75 (83.00 / 112.00)	22.98 (5.20 / 39.80)	23.00 (19.10 / 26.90)	27.01 (19.22 / 31.12)	27.80 (24.72 / 29.94)	1136 / 538 / 441	41.38 / 19.60 / 16.07

ES1599A<br>(rural)	27	94.25<br>(43.00 / 180.00)	94.00<br>(80.00 / 106.00)	20.11<br>(7.00 / 37.20)	20.00<br>(17.20 / 22.60)	22.81<br>(18.62 / 25.14)	23.50<br>(21.30 / 24.50)	916 / 542 / 342	33.37 / 19.74 / 12.46
**4-HA - 8 rural, 0 urban and 0 suburban (total: 8) stations**									
DESN053<br>(rural)	1	111.39<br>(49.74 / 230.00)	109.00<br>(93.66 / 127.00)	13.36<br>(-6.40 / 30.60)	13.50<br>(9.60 / 17.50)	17.64<br>(9.30 / 21.90)	18.70<br>(15.00 / 20.22)	1706 / 546 / 519	62.15 / 19.89 / 18.91
**5-NE - 10 rural, 6 urban and 1 suburban (total: 17) stations**									
GB0033R<br>(rural)	1	75.67<br>(24.10 / 158.00)	73.64<br>(63.91 / 87.31)	16.50<br>(2.50 / 28.60)	16.60<br>(14.00 / 18.80)	18.75<br>(13.50 / 22.00)	19.50<br>(17.00 / 20.40)	193 / 510 / 80	7.03 / 18.58 / 2.91
GB0567A<br>(urban)	2	66.38<br>(16.00 / 188.00)	64.00<br>(54.00 / 76.67)	16.92<br>(4.30 / 29.00)	17.10<br>(14.70 /19.10)	18.92<br>(13.70 / 21.40)	19.80<br>(17.40 / 20.50)	87 / 524 / 46	3.17 / 19.09 / 1.68
IE0001R<br>(suburban)	7	74.03<br>(1.85 / 89.40)	72.20<br>(60.40 / 89.40)	16.15<br>(7.00 / 28.50)	16.40<br>(14.40 / 17.80)	17.50<br>(13.50 / 19.10)	18.50<br>(16.10 / 18.70)	341 / 512 / 116	12.42 / 18.65 / 4.23
**6-SE - 0 rural, 9 urban and 8 suburban (total: 17) stations**									
ES1666A<br>(urban)	1	103.33<br>(29.00 / 233.00)	101.00<br>(90.00 / 114.00)	26.49<br>(11.30 / 39.80)	27.40<br>(23.40 / 29.90)	28.65<br>(21.30 / 32.50)	29.70<br>(25.84 / 31.70)	1287 / 495 / 276	46.89 / 18.03 / 10.05
ES1215A<br>(suburban)	2	101.01<br>(33.00 / 195.00)	100.00<br>(91.00 / 110.00)	29.33<br>(10.60 / 42.30)	30.10<br>(26.20 / 33.10)	31.84<br>(24.60 / 35.00)	33.00<br>(29.10 / 35.00)	1248 / 499 / 291	45.46 / 18.18 / 10.60

Note. The 15 selected representative stations per o-t-region are shown. O-t-regions are framed in the table by dashed lines. The total number as well as the number of stations per type are given as well. Position values refer to the proximity of a representative station to its respective cluster centroid based on the ranking of all region-specific stations with respect to their Euclidean distances to their cluster centroid (for example, considering the ranking of stations in 3-CW based on all computed Euclidean distances, station FR07004 is on position 2 of 33 being the second closest to its region's centroid). Please keep in mind that the shown positions also depend on the number of stations present in a region for each station type (i.e., ES1599A being the only rural station in 3-CW). Mean, median, minimum, maximum as well as 25% and 75% quantiles based on the months from April to September across all years are depicted for both target variables. The absolute and relative numbers of o-, t- and o-t-events with respect to the total number of days in the base period from 2004 to 2018 are shown as well.

Table S6 *Association of Weather Types with Event Days*

Region events		WT1	WT2	WT3	WT4	WT5	WT6	WT7	WT8	WT9	Total
						**1-CE**					
Ozone	n	135	165	102	162	222	153	155	202	151	1447
	%	9.33	11.4	7.05	11.2	**15.34**	10.57	10.71	13.96	10.44	
Temperature	n	48	40	46	62	108	84	69	107	96	660
	%	7.27	6.06	6.97	9.39	16.36	12.73	10.45	**16.21**	14.55	
Ozone - Temperature	n	43	39	31	61	105	75	66	102	87	609
	%	7.06	6.4	5.09	10.02	**17.24**	12.32	10.84	**16.75**	14.29	
						**2-CN**					
Ozone	n	59	84	39	104	194	101	144	154	99	978
	%	6.03	8.59	3.99	10.63	**19.84**	10.33	14.72	**15.75**	10.12	
Temperature	n	41	46	17	61	137	87	102	121	103	715
	%	5.73	6.43	2.38	8.53	**19.16**	12.17	14.27	**16.92**	14.41	
Ozone - Temperature	n	25	40	10	54	123	60	92	102	68	574
	%	4.36	6.97	1.74	9.41	**21.43**	10.45	**16.03**	**17.77**	11.85	
						**3-CW**					
Ozone	n	232	170	107	210	229	150	182	231	180	1691
	%	13.72	10.05	6.33	12.42	13.54	8.87	10.76	13.66	10.64	
Temperature	n	112	72	73	77	162	105	70	130	107	908
	%	12.33	7.93	8.04	8.48	**17.84**	11.56	7.71	14.32	11.78	

Ozone - Temperature	n	98	51	37	63	135	83	64	121	88	740
	%	13.24	6.89	5	8.51	**18.24**	11.22	8.65	**16.35**	11.89	
**4-HA**											
Ozone	n	179	204	142	193	235	186	158	226	183	1706
	%	10.49	11.96	8.32	11.31	13.77	10.9	9.26	13.25	10.73	
Temperature	n	51	47	44	54	68	80	31	82	89	546
	%	9.34	8.61	8.06	9.89	12.45	14.65	5.68	**15.02**	**16.3**	
Ozone - Temperature	n	50	43	41	53	65	75	30	79	83	519
	%	9.63	8.29	7.9	10.21	12.52	14.45	5.78	**15.22**	**15.99**	
**5-NE**											
Ozone	n	232	170	107	210	229	150	182	231	180	908
	%	13.72	10.05	6.33	12.42	13.54	8.87	10.76	13.66	10.64	
Temperature	n	112	72	73	77	162	105	70	130	107	1691
	%	12.33	7.93	8.04	8.48	**17.84**	11.56	7.71	14.32	11.78	
Ozone - Temperature	n	98	51	37	63	135	83	64	121	88	740
	%	13.24	6.89	5	8.51	**18.24**	11.22	8.65	**16.35**	11.89	
**6-SE**											
Ozone	n	260	173	121	222	187	135	192	206	163	1659
	%	**15.67**	10.43	7.29	13.38	11.27	8.14	11.57	12.42	9.83	
Temperature	n	130	72	89	74	68	61	57	78	69	698
	%	**18.62**	10.32	12.75	10.6	9.74	8.74	8.17	11.17	9.89	

Ozone - Temperature	n	94	42	41	47	45	28	42	48	45	432
	%	**21.76**	9.72	9.49	10.88	10.42	6.48	9.72	11.11	10.42	

Note. An overview of the association of weather types with o-, t- as well as o-t- events in the o-t-season from April to September across all years from 2004 to 2018 is given. Shown are the absolute (n) and relative (%) numbers of event days by considering all representative stations of an o-t-region (from 1-CE to 6-SE). Event days of a type which occurred simultaneously at multiple stations of a region are only counted once. Relative contributions of a weather type to an event above 15% are marked in bold. Note that minor differences in the depicted percentage values are due to rounding.

Table S7 *Projected Ensemble Changes [%]*

Station	SSP245						SSP370					
	2041-2060			2081-2100			2041-2060			2081-2100		
	median	min	max	median	min	max	median	min	max	median	min	max
DEST066	33.06	9.60<br>FGOALS-g3	86.92<br>CanESM5	56.32	14.40<br>FGOALS-g3	122.62<br>CanESM5	41.22	15.19<br>MPI-ESM1-2-HR	138.75<br>CanESM5	78.05	17.68<br>FGOALS-g3	332.92<br>CanESM5
DEBB066	36.40	10.21<br>INM-CM5-0	102.49<br>CanESM5	57.21	18.04<br>FGOALS-g3	138.63<br>CanESM5	44.01	14.93<br>MPI-ESM1-2-HR	140.85<br>CanESM5	90.37	19.26<br>FGOALS-g3	358.69<br>CanESM5
DEST002	39.64	13.89<br>INM-CM5-0	100.00<br>CanESM5	65.52	18.65<br>FGOALS-g3	148.57<br>CanESM5	45.94	17.00<br>MPI-ESM1-2-HR	168.37<br>CanESM5	93.45	22.92<br>FGOALS-g3	419.39<br>CanESM5
NL00807	39.66	15.40<br>MPI-ESM1-2-HR	109.06<br>CanESM5	75.88	19.56<br>FGOALS-g3	147.46<br>CanESM5	62.66	28.47<br>FGOALS-g3	141.34<br>CanESM5	115.45	35.41<br>FGOALS-g3	421.79<br>CanESM5
DEHH047	49.37	20.85<br>INM-CM5-0	108.99<br>CanESM5	74.02	31.33<br>FGOALS-g3	148.92<br>CanESM5	48.25	31.90<br>MPI-ESM1-2-HR	153.45<br>CanESM5	99.15	25.83<br>FGOALS-g3	445.98<br>CanESM5
DEHB002	42.24	19.71<br>INM-CM5-0	112.95<br>CanESM5	79.99	31.15<br>FGOALS-g3	160.71<br>CanESM5	57.38	35.03<br>IPSL-CM6A-LR	170.68<br>CanESM5	113.46	26.37<br>FGOALS-g3	500.00<br>CanESM5
FR26010	48.51	20.45<br>INM-CM5-0	112.46<br>IPSL-CM6A-LR	76.92	12.13<br>FGOALS-g3	163.67<br>IPSL-CM6A-LR	58.88	27.36<br>FGOALS-g3	158.69<br>CanESM5	127.66	61.15<br>MPI-ESM1-2-HR	330.52<br>CanESM5
FR07004	53.68	24.08<br>INM-CM5-0	130.97<br>IPSL-CM6A-LR	82.83	29.31<br>FGOALS-g3	199.56<br>IPSL-CM6A-LR	75.99	29.58<br>MPI-ESM1-2-HR	162.84<br>CanESM5	159.80	54.63<br>MPI-ESM1-2-HR	361.70<br>IPSL-CM6A-LR
ES1599A	49.47	18.39<br>INM-CM5-0	203.55<br>IPSL-CM6A-LR	110.45	41.10<br>FGOALS-g3	273.76<br>IPSL-CM6A-LR	100.39	41.94<br>INM-CM5-0	194.90<br>CanESM5	179.02	81.36<br>INM-CM5-0	509.18<br>CanESM5
DESN053	32.57	10.70<br>FGOALS-g3	78.05<br>CanESM5	58.35	8.72<br>FGOALS-g3	122.92<br>IPSL-CM6A-LR	47.12	8.24<br>MPI-ESM1-2-HR	160.57<br>CanESM5	94.46	26.40<br>FGOALS-g3	323.58<br>CanESM5

Note. Ensemble median changes [%] regarding the number of days with o-t-events between the periods 2041-2060 (mid-century) as well as 2081-2100 (end-century) compared to the historical ESM period 1995-2014 for all representative stations of 1-CE, 2-CN, 3-CW and 4-HA are shown. O-t-regions are separated by dashed lines. Minimum and maximum changes with respective ESM names in brackets based on single ESM projections are given as well. SSP245 and SSP370 scenario assumptions are considered.

Table S8 *Region-Specific MT Anomaly Mean Warmings*

**O-t-region**	**SSP245**		**SSP370**	
	**2041-2060**	**2081-2100**	**2041-2060**	**2081-2100**
1-CE	1.22 (0.31 / 3.41)	2.03 (0.77 / 3.97)	1.58 (0.49 / 3.56)	3.26 (1.64 / 4.96)
2-CN	1.20 (0.40 / 3.11)	1.99 (0.82 / 3.60)	1.52 (0.59 / 3.35)	3.14 (1.56 / 5.02)
3-CW	1.74 (0.42 / 3.39)	2.67 (0.65 / 4.68)	2.06 (0.65 / 4.09)	4.15 (1.86 / 6.25)
4-HA	1.34 (0.24 / 3.63)	2.19 (0.55 / 4.26)	1.70 (0.23 / 3.62)	3.51 (1.50 / 5.16)

Note. Based on o-t-seasonal daily ESM data, mean warmings per time window (2041-2060 and 2081-2100) are calculated for the two SSP scenarios in the chosen CMIP6 ensemble for all Central European o-t-regions (from 1-CE to 4-HA). Warmings are expressed by MT anomalies and are shown per region. Averages across daily time series data of all representative stations of an o-t-region are considered to get one region-specific daily time series data. Means of this region-specific daily historical ESM data from 1995 to 2014 build the baseline to define MT anomalies. The ensemble warming anomalies per time window are calculated based on this baseline mean across all eight chosen ESM per scenario and o-t-region. While the depicted values represent the multi-model mean results of all eight ESM, numbers in brackets refer to the minimum and maximum values grounded on one specific ESM.

Figure S1
Relationship between MDA8O3 and TX for all representative stations and o-t-regions. All daily observations from April to September across all years from 2004-2018 are considered. GAM between both target variables are shown to highlight the linkage between both variables (blue line). Grey shadings illustrate the used confidence interval (0.95). Red horizontal lines illustrate the WHO guideline of 100 µg/m^3. Vertical lines show the 80% quantile of all respective observed TX values across all months and years. Note that representative station DEST002 of 1-CE is not depicted, again.

1-CE

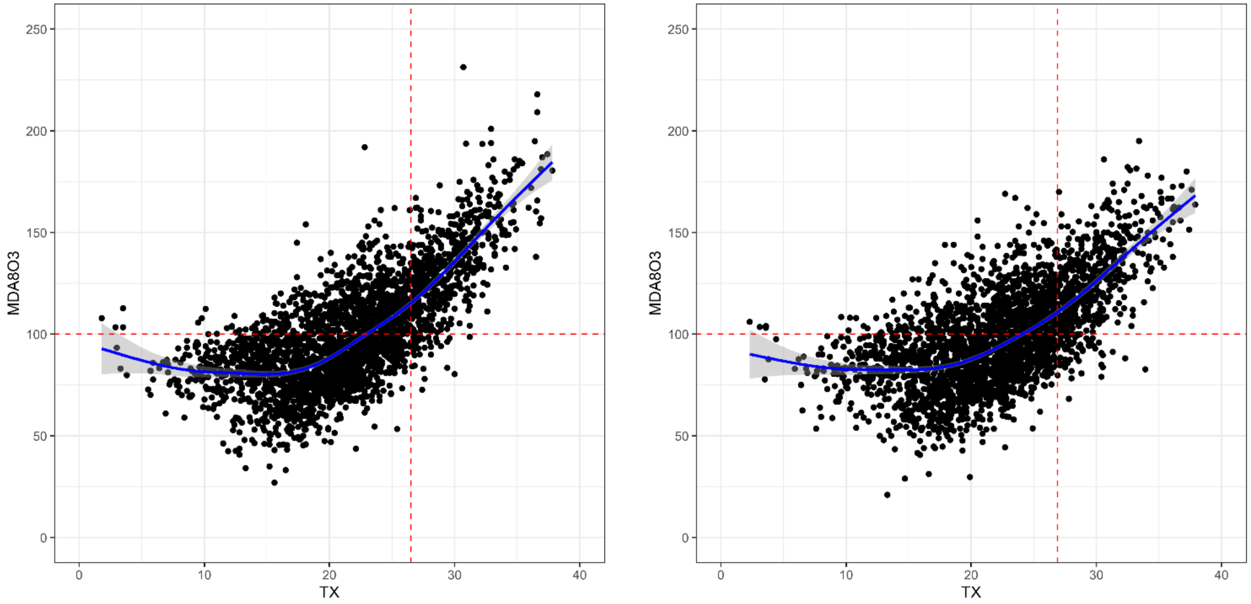

a) DEST066 b) DEBB066

2-CN

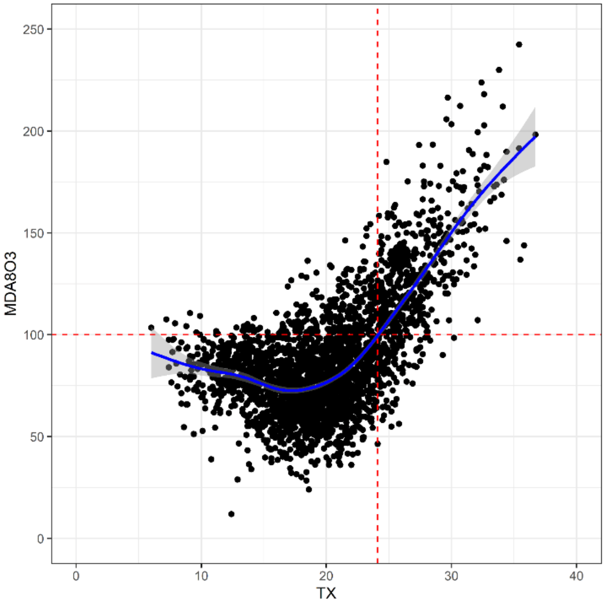

c) NL00807

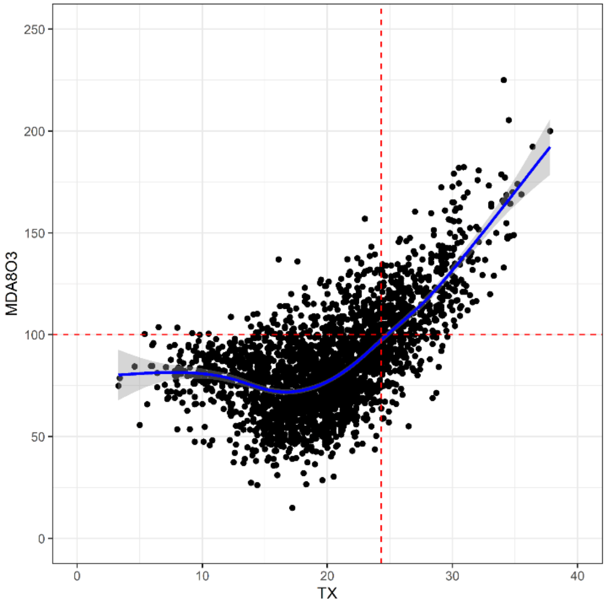

d) DEHH047

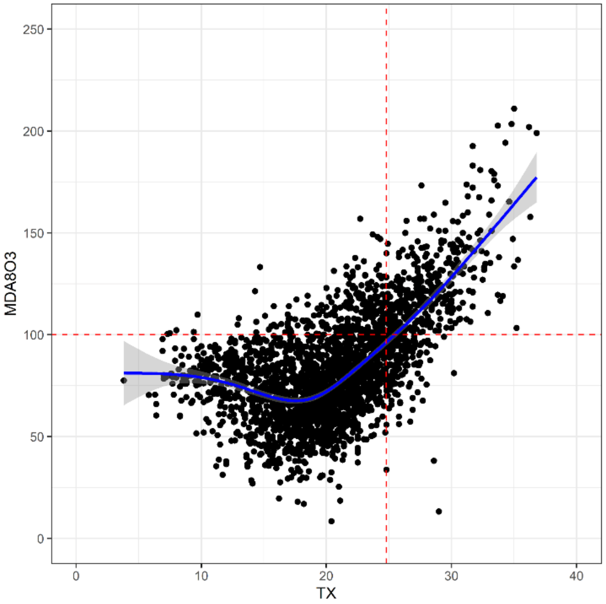

e) DEHB002

3-CW

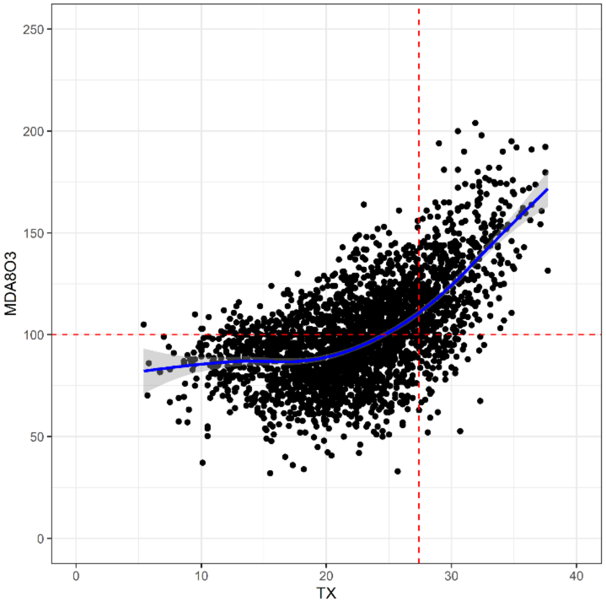

f) FR26010

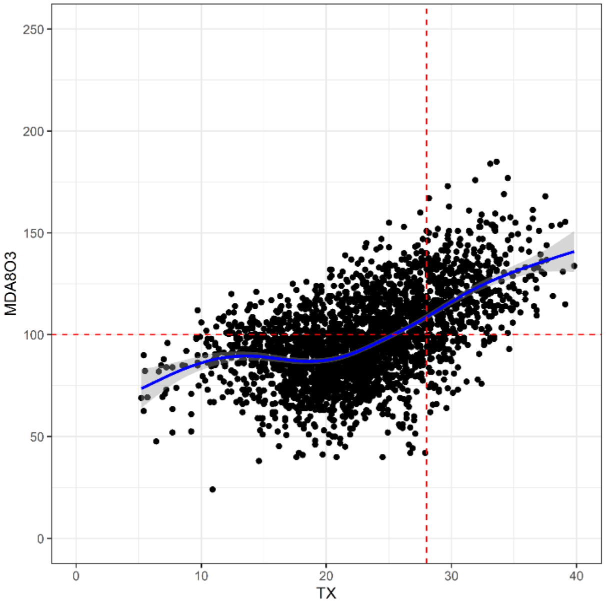

g) FR07004

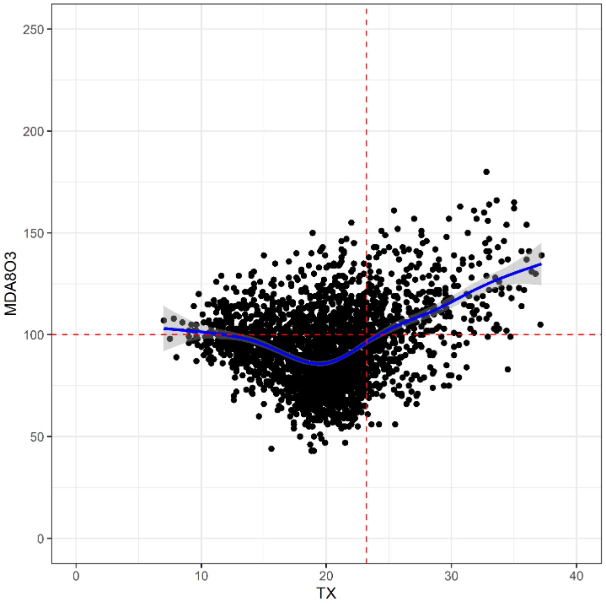

h) ES1599A

4-HA

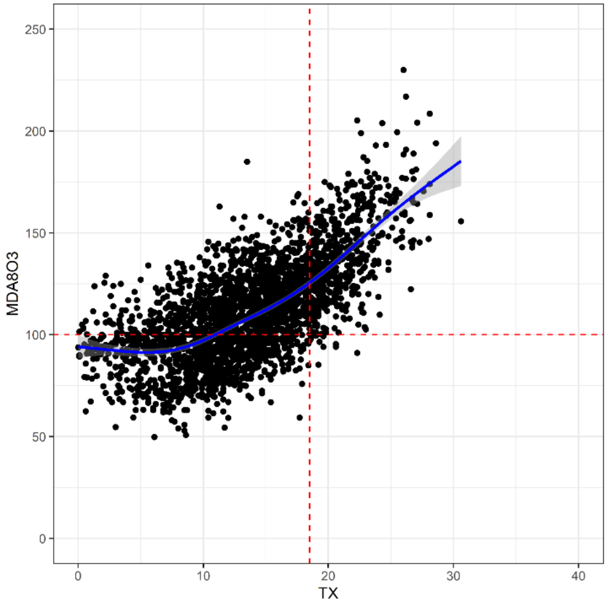

i) DESN053

5-NE

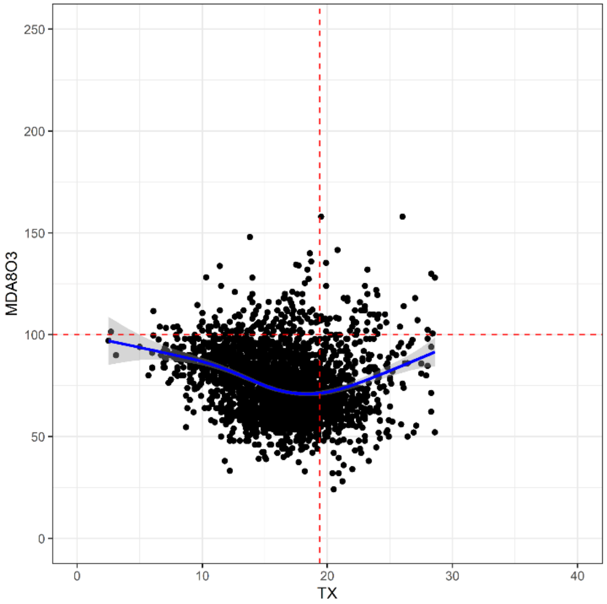

j) GB0033R

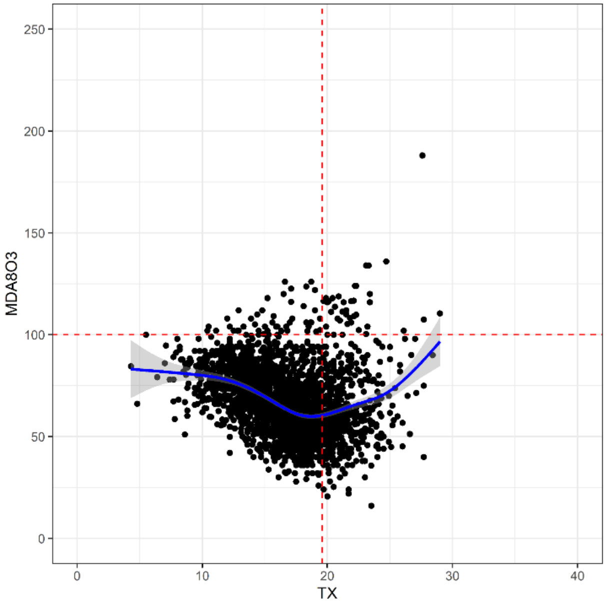

k) GB0567A

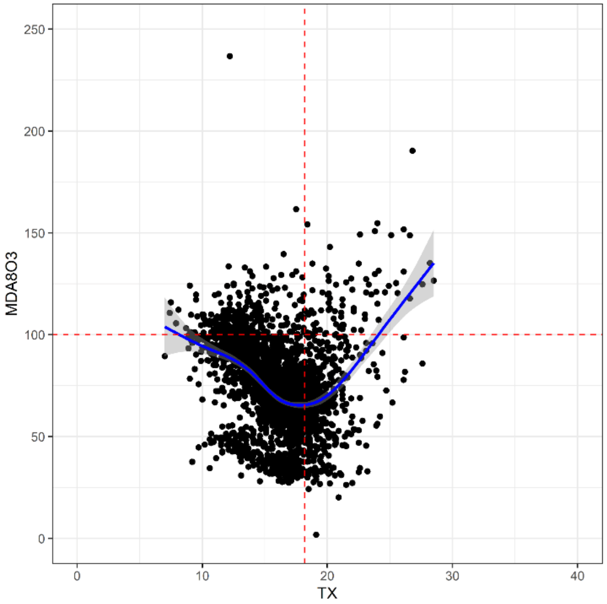

l) IE0001R

6-SE

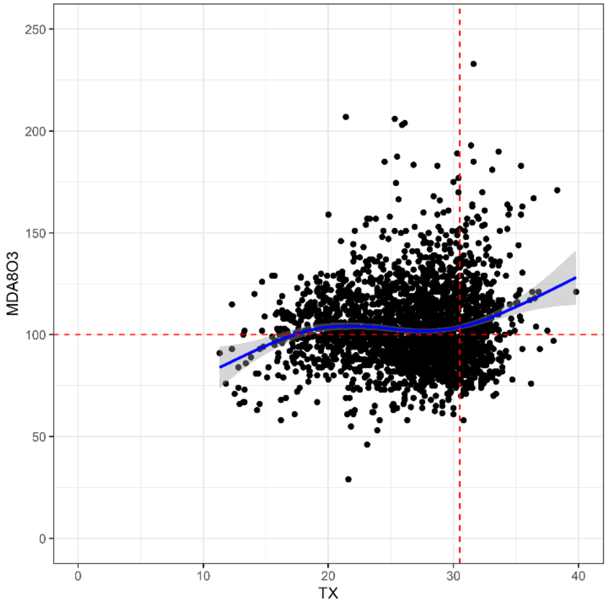

m) ES1666A

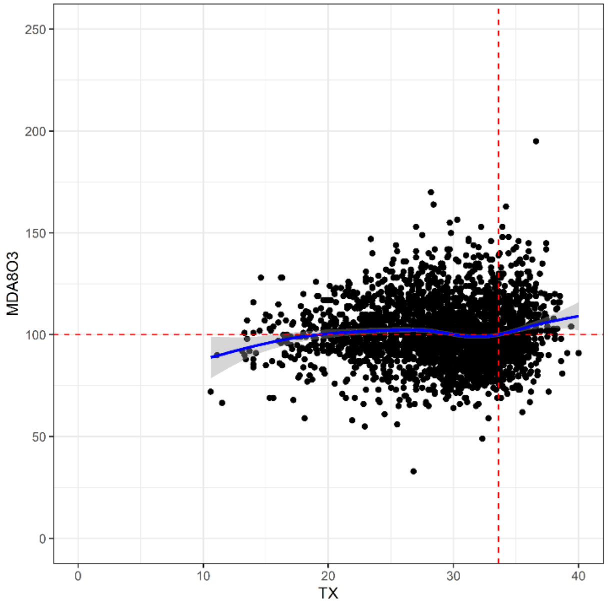

n) ES1215A

Figure S2

Overview of the final 9 synoptic weather types in Europe created by the applied weather classification scheme using the SOM algorithm. Classification grounds on the o-t-season from 2004 to 2018. Each weather type is based one node of the SOM. The respective patterns shown are the MSLP- “weights” of each node. As the array of nodes self-organize into a pattern with more similar nodes being into closer proximity and more dissimilar nodes further away, simple clusters can be detected. Consequently, weather types WT1 (upper left corner) to WT9 (bottom right corner) are presented. Atmospheric pressure levels are shown in hPa.

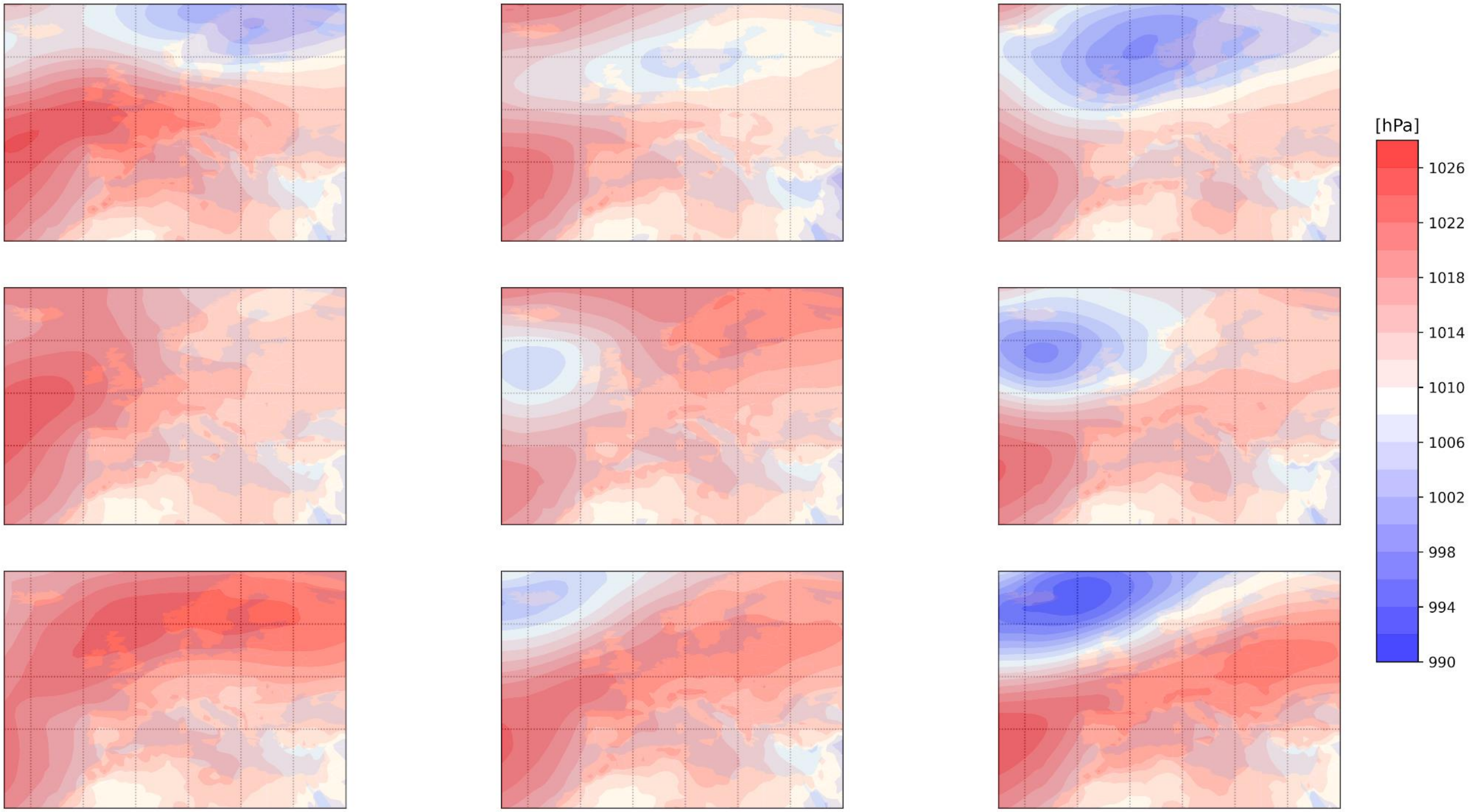

Supplement: Supplementary file 1 — Supporting Information S1 [file GH2-6-e2021GH000561-s001.pdf]
